# Supplementary material for: Combined Systematic Review and Transcriptomic Analyses of Mammalian Aquaporin Classes 1 to 10 as Biomarkers and Prognostic Indicators in Diverse Cancers
Source: Cancers (Basel). 2020 Jul 15;12(7):1911. doi: 10.3390/cancers12071911 (PMC7409285; doi:10.3390/cancers12071911)
Supplement: Supplementary file 1 [file cancers-12-01911-s001.pdf]

cancers

MDPI

**Supplementary Table S1.** Extracted Data.

| No. | First author | Study type | Method | Tumour type | Aquaporin type | Expression/ function or both |
|---|---|---|---|---|---|---|
| [1] | Abdelrahman, A. E 2018 | Biopsy | Immunohistochemistry | Ovarian cancer | AQP5 | Expression |
| [2] | Allory, Y. 2008 | Biopsy | Immunohistochemistry | Renal cancer | AQP1 | Expression |
| [3] | Angelico, G. 2018 | Biopsy | Immunohistochemistry | Pleural Mesothelioma | AQP1 | Expression |
| [4] | Angelico, G. 2018 | Biopsy | Immunohistochemistry | Pleural Mesothelioma | AQP1 | Expression |
| [5] | Aras, Y. 2016 | In vitro | Immunohistochemistry | Brain cancer | AQP4 | Expression |
| [6] | Arif, M. 2018 | In vitro | Small interfering RNA<br>RT PCR<br>Fluorescent cell swelling assay<br>Cellular proliferation assay<br>Cellular migration assays<br>Cellular invasion assay<br>Cellular adhesion assay | Breast cancer | AQP3 | Expression and function |
| [7] | Bellezza, G. 2017 | Biopsy | Immunohistochemistry | Lung cancer | AQP1,5 | Expression |
| [8] | Ben, Y. 2008 | In vitro | RT PCR<br>Immunohistochemistry | Lung cancer | AQP3,5 | Expression |
| [9] | Bhattacharjee, H. 2004 | In vitro | RT PCR<br>Up take assay | Leukemia | AQP9 | Expression and function |
| [10] | Breyer, J. 2017 | Biopsy | Immunohistochemistry | Urothelial cancer | AQP3 | Expression |
| [11] | Cagini, L. 2017 | Biopsy | RT PCR | Lung cancer | AQP1<br>AQP5 | Expression |
| [12] | Cao, X. C. 2013 | In vitro | Wound healing assay<br>Western blot<br>Small interfering RNA | Breast cancer | AQP3 | Expression and function |
| [13] | Capoccia, 2015 | In vitro | Western blot<br>Wound healing assay<br>DNA fragmentation assay<br>Cell proliferation and survival assays<br>Apoptotic cell staining | Brain cancer | AQP4 | Expression and function |
| [14] | Chae, Y. K. 2008 | In vitro | Small interfering RNA<br>RT PCR | Leukemia | AQP5 | Expression and function |

| | | | | | | |
|---|---|---|---|---|---|---|
| | | | Immunohistochemistry<br>Immunoblotting<br>Flow cytometry<br>Cytogenetic response | | | |
| [15] | Chae, Y. K. 2008 | Biopsy<br>In vitro | Tissue microarrays<br>Pull-down assays<br>Invasion assay<br>Immunoprecipitation<br>Immunohistochemistry<br>Fluorescence in situ hybridization | Lung cancer | AQP5 | Expression and function |
| [16] | Chae, Y. S. 2015 | Biopsy | Tissue array<br>Immunohistochemistry | Breast cancer | AQP3 | Expression |
| [17] | Chang, H. 2014 | Biopsy | Western blot<br>RT PCR<br>Immunohistochemistry | Cervical cancer | AQP8 | Expression |
| [18] | Chang, H. 2014 | In vitro | Wound healing assay<br>Western Blot<br>Immunohistochemistry | Oesophageal cancer | AQP8 | Expression and function |
| [19] | Chau, D. 2015 | In vitro | Western blot<br>Small interfering RNA<br>RT PCR<br>Methylation-specific PCR<br>Cytotoxicity assay<br>Apoptosis assay | Leukemia | AQP9 | Expression and Function |
| [20] | Chen, C. 2017 | In vitro | Wound healing assay<br>Transwell migration<br>Invasion assays<br>Cytotoxicity assay | Colon cancer | AQP5 | Expression and function |
| [21] | Chen, G. 2018 | In vitro<br>In vivo | Western blot<br>Tumour growth in vivo<br>RT PCR<br>Invasion assays<br>Immunohistochemistry<br>Cell proliferation | Liver cancer | AQP3 | Expression and function |
| [22] | Chen, J. 2004 | In vitro | RT PCR | Lung cancer | AQP 1,3,4,5 | Expression |

| | | | | | | |
|---|---|---|---|---|---|---|
| [23] | Chen, J. 2014 | Biopsy<br>In vitro | Western blot<br>Transwell migration<br>Invasion assays<br>Small interfering RNA<br>RT PCR<br>Immunohistochemistry<br>Immunofluorescence assays<br>Cell proliferation assays | Gastric cancer | AQP2 | Expression and Function |
| [24] | Chen, J. 2015 | In vitro | Wound healing assay<br>Western blot<br>Small interfering RNA<br>RT PCR<br>MMP-3 ELISA assay<br>Invasion assays<br>cDNA microarray | Prostate cancer | AQP2 | Expression and function |
| [25] | Chen, L. 2017 | Biopsy<br>In vitro | Western blot<br>Small interfering RNA<br>RT PCR<br>Immunohistochemistry<br>Immunofluorescence assays<br>Flow cytometry assay<br>Cellular TAG level detection assay | Gastric cancer | AQP3 | Expression and function |
| [26] | Chen, Q. 2016 | In vitro | Wound healing assay<br>Western blot<br>Invasion assays<br>Small interfering RNA<br>RT PCR<br>Immunohistochemistry<br>Cell Proliferation Assay<br>Cell apoptosis assay. | Prostate cancer | AQP8 | Expression and function |
| [27] | Chen, Q 2018 | In vitro | Wound healing assay<br>Western blot<br>Invasion assays<br>Small interfering RNA<br>RT PCR<br>Cell cycle distribution assay<br>Cell apoptosis assay | Prostate cancer | AQP3 | Expression and function |

| | | | | | | |
|---|---|---|---|---|---|---|
| [28] | Chen, R. 2012 | Biopsy | RT PCR<br>Immunohistochemistry | Cervical cancer | AQP 1,3,8 | Expression |
| [29] | Chen, R. 2014 | Biopsy | RT PCR<br>Immunohistochemistry | Cervical cancer | AQP 1,3 | Expression |
| [30] | Chen, X. 2014 | In vitro | RT PCR<br>MTT assay | Ovarian cancer | AQP1 to 10 | Expression and function |
| [31] | Chen, X. 2015 | In vitro | Western blot<br>Cell proliferation assay<br>Small interfering RNA<br>RT-qPCR | Ovarian cancer | AQP5 | Expression and function |
| [32] | Chen, X. F. 2016 | Biopsy | Western blot<br>qRT PCR<br>Immunohistochemistry | Liver cancer | AQP 3,7,9 | Expression |
| [33] | Chen, Y. 2017 | In vitro | Western blot<br>qRT PCR<br>Invasion<br>Cell viability assay<br>Cell migration assay<br>Cell cycle analysis | Brain cancer | AQP 4 | Expression and function |
| [34] | Chen, Y. 2003 | Biopsy | ISH of Tissue Microarrays | Cervical cancer | AQP5 | Expression |
| [35] | Chen Y. 2006 | In vitro | Western Blots<br>RT PCR<br>Immunohistochemistry<br>CT scan<br>MRI | Brain cancer | AQP1 | Expression |
| [36] | Chen Z. 2011 | Biopsy | Western Blot<br>Water permeability assay<br>qRT PCR<br>Migration assay<br>Invasion assay<br>Colony forming test<br>Cellular regulatory volume | Lung cancer | AQP5 | Expression and function |
| [37] | Deb, P. 2012 | In vitro | Immunohistochemistry | Brain cancer | AQP1 | Expression |
| [38] | Ding, T. 2011 | Biopsy<br>In vivo | Western Blot<br>Small interfering RNA<br>Scratch assay<br>Osmotic fragility | Brain cancer | AQP4 | Expression and function |

| | | | | | | |
|---|---|---|---|---|---|---|
| | | | Invasion assay<br>F-actin measurement<br>Cytotoxicity assay<br>Chemotaxis and chemo kinesis assays<br>Aggregation assay<br>Adhesion assay | | | |
| [39] | Ding, T. 2013 | Biopsy<br>In vivo | Western Blot<br>Tumorigenicity Assay<br>Small interfering RNA<br>Osmotic fragility<br>MTT assay<br>Immunohistochemistry<br>Colonies formation assay<br>Apoptosis assay | Brain cancer | AQP4 | Expression and function |
| [40] | Dong, X. 2016 | Biopsy | Western Blot<br>Small interfering RNA<br>Immunohistochemistry<br>Cell proliferation assays | Gastric cancer | AQP3 | Expression and function |
| [41] | Dorward, H. S. 2016 | Biopsy<br>In vitro | Wound healing assay<br>Western Blot<br>Immunohistochemistry<br>qPCR<br>Invasion assay<br>Immunofluorescence<br>Cell proliferation assay<br>Angiogenesis assay | Colon cancer | AQP1 | Expression and function |
| [42] | Dou, R. 2013 | In vitro | MicroRNA data analysis<br>Gene expression data analysis | Colon cancer | AQP9 | Expression |
| [43] | Driml, J. 2013 | Biopsy<br>In vivo | VEGFA ELISA<br>Small interfering RNA<br>Matrigel VM Assay<br>qRT PCR<br>Mouse Xenograft MM Model | Malignant mesothelioma | AQP1 | Expression and function |
| [44] | Driml, J. 2016 | In vitro | Immunohistochemistry | Malignant mesothelioma | AQP1 | Expression |
| [45] | Dua, R. K. 2010 | In vitro | Immunohistochemistry | Brain cancer | AQP4 | Expression |
| [46] | El Hindy, N. 2013 | In vitro | Immunohistochemistry | Brain cancer | AQP1 | Expression |

| | | | | | | |
|---|---|---|---|---|---|---|
| [47] | El Hindy, N. 2013 | In vitro | Secreted Alkaline Phosphatase (SEAP) Assay<br>qRT PCR<br>MGMT-Promoter Methylation Analysis<br>Immunohistochemistry | Brain cancer | AQP1 | Expression |
| [48] | Endo, M. 1999 | In vivo | Immunofluorescence | Brain cancer | AQP1 | Expression |
| [49] | Esghaei, M. 2018 | Biopsy | MTT assay<br>Immunofluorescence | Colon cancer | AQP5 | Expression and function |
| [50] | Evans, J. 2017 | Biopsy | Western blot<br>MTT assay<br>Small interfering RNA | Lung cancer | AQP11 | Expression |
| [51] | Ewelt, C. 2012 | In vitro | Immunofluorescence | Brain cancer | AQP1<br>AQP4 | Expression |
| [52] | Fallier-Becker, P. 2013 | Biopsy<br>In vivo | Western blot<br>RT-PCR<br>Immunocytochemistry | Brain cancer | AQP4 | Expression |
| [53] | Fossdal, G. 2012 | Biopsy<br>In vitro<br>In vivo | Western blot<br>qPCR<br>Immunocytochemistry | Brain cancer | AQP9 | Expression |
| [54] | Georges, J. 2011 | In vivo<br>In vitro | Western blot<br>RT PCR | Brain cancer | AQP1 | Expression |
| [55] | Guan, Y. 2018 | In vitro | Western blot<br>Small interfering RNA<br>MTT Test<br>qRT PCR<br>Migration assay<br>Invasion assay<br>Apoptosis | Brain cancer | AQP1 | Expression and function |
| [56] | Guo, K. 2015 | Biopsy | Western blot<br>Small interfering RNA<br>MTT assay<br>Migration assay | Lung cancer | AQP5 | Expression and function |
| [57] | Guo, X. 2013 | In vitro | Immunocytochemistry | Liver cancer | AQP3<br>AQP5 | Expression |
| [58] | Hara-Chikuma, M. 2008 | In vivo | Water and glycerol permeability assay | Skin cancer | AQP3 | Expression and function |

| | | | | | | |
|---|---|---|---|---|---|---|
| | | | RT PCR<br>Cell proliferation assay<br>Cell differentiation assay<br>Cell apoptosis assay<br>ATP production assay | | | |
| [59] | Hara-Chikuma, M. 2016 | Biopsy | RT PCR<br>Immunofluorescence<br>Immunobloting<br>Cellular H2O2 analysis | Skin cancer<br>Lung cancer | AQP3 | Expression and function |
| [60] | Hayashi, 2007 | In vitro | Western blot<br>Lactate Dehydrogenase Assay<br>Lactate Assay<br>Immunohistochemistry<br>Cathepsin B Enzyme Activity Assay | Brain cancer | AQP1 | Expression and function |
| [61] | He, Z. 2017 | Biopsy<br>In vivo | Western blot<br>Small interfering RNA<br>qRT PCR<br>Cell migration assay<br>Cell invasion assay | Liver cancer | AQP5 | Expression and function |
| [62] | Hindy, N. E. 2013 | In vivo | Immunohistochemistry | Brain cancer | AQP1 | Expression |
| [63] | Hoque, M. O. 2006 | Biopsy<br>In vitro | Western blot<br>Immunohistochemistry<br>Terminal dUTP Nick-End Labeling (TUNEL) Assay<br>Focus Formation Assay<br>RT PCR<br>In vitro growth rate<br>ERK Activity assay | Lung cancer | AQP1 | Expression and function |
| [64] | Hu, H. 2005 | In vivo | Immunohistochemistry | Brain cancer | AQP4 | Expression |
| [65] | Huang, D. 2017 | Biopsy<br>In vitro<br>In vivo | Western blot<br>Cell growth curve assay<br>qRT PCR<br>Immunohistochemistry<br>Flow cytometry | Colon cancer | AQP9 | Expression and function |
| [66] | Huang, X. 2017 | Biopsy | Western blot<br>Small interfering RNA | Pancreatic cancer | AQP3 | Expression and function |

| | | | | | | |
|---|---|---|---|---|---|---|
| | | | Luciferase reporter assay<br>Cell proliferation and apoptosis assay | | | |
| [67] | Huang, Y. 2009 | In vivo | qRT PCR | Renal cancer | AQP1 | Expression |
| [68] | Huang, Y. 2010 | Biopsy | Western blot<br>Small interfering RNA<br>Migration assay<br>Cell proliferation assay | Gastric cancer | AQP3 | Expression and function |
| [69] | Huang, Y. H.2013 | Biopsy<br>In vivo | Western blot<br>RT PCR<br>MTT assay<br>Migration assay<br>Immunohistochemistry<br>Colony formation assay | Gastric cancer | AQP5 | Expression and function |
| [70] | Huang, Y. T. 2015 | In vivo<br>Biopsy | Western blot<br>Small interfering RNA<br>RT PCR<br>Migration assay<br>Luciferase reporter assay<br>Invasion assay<br>Immunohistochemistry<br>Bioinformation and chromatin immunoprecipitation (ChIP) analyses | Breast cancer | AQP3 | Expression and function |
| [71] | Hwang, I. 2012 | In vivo | Western blot<br>Immunofluorescence | Prostate cancer | AQP1,3,9 | Expression |
| [72] | Imaizumi, H. 2018 | In vivo | Immunohistochemistry | Colon cancer | AQP1 | Expression |
| [73] | Imrédi, E. 2018 | In vivo | Immunohistochemistry | Skin cancer | AQP1 | Expression |
| [74] | Imrédi, E. 2016 | In vivo | Immunohistochemistry | Skin cancer | AQP1 | Expression |
| [75] | Iriyama, N. 2013 | Biopsy<br>In vivo | RT PCR<br>Apoptosis assay | Leukemia | AQP9 | Expression and function |
| [76] | Ishimoto, S. 2012 | Biopsy<br>In vivo | Immunohistochemistry<br>Western blot<br>Small interfering RNA<br>Cell survival assay<br>Adhesion | Tongue cancer | AQP3<br>AQP5 | Expression and function |
| [77] | Ismail, M. 2009 | Biopsy | Western blot | Prostate cancer | AQP3 | Expression |

| | | | | | | |
|---|---|---|---|---|---|---|
| | | | Small interfering RNA<br>RT PCR<br>Immunofluorescence<br>Cryoinjury assay | | | |
| [78] | Isokpehi, R. D. 2015 | Biopsy | Gene analysis | Brain cancer | All AQPs | Expression |
| [79] | Jagirdar, R. 2013 | Gene database | BioGRID analysis | Pleural mesothelioma | AQP1 | Expression |
| [80] | Jagirdar, R. M. 2016 | Biopsy | Western blot<br>Sphere formation assay<br>RT PCR<br>Cell migration assay<br>Cell adhesion | Pleural mesothelioma | AQP1 | Expression and function |
| [81] | Jelen, S. 2013 | Biopsy<br>In vivo | Immunofluorescence | Brain cancer | AQP9 | Expression |
| [82] | Ji, C. 2008 | Biopsy | Western Blot<br>Small interfering RNA<br>Phagokinetic track motility assay | Ovarian cancer | AQP3 | Expression and function |
| [83] | Jia, B. 2018 | Biopsy | Western blot<br>Small interfering RNA RT PCR<br>Invasion assay<br>CCk-8 assay | Breast cancer | AQP5 | Expression and function |
| [84] | Jiang, B. 2014 | Biopsy<br>In vitro<br>In vivo | Western blot<br>TUNEL assay<br>Tumorigenicity<br>qRT PCR<br>Luciferase assay<br>Immunohistochemistry<br>Cell migration assay<br>Cell invasion assay<br>Cell apoptosis assay<br>Caspase-3 activity assay<br>Colony formation assay<br>5-ethynyl-2 -deoxyuridine (EdU) assay | Gastric cancer | AQP3 | Expression and function |
| [85] | Jo, Y. M. 2016 | Biopsy | Immunohistochemistry | Lung cancer | AQP5 | Expression |
| [86] | Jung, H. J. 2011 | Biopsy | RT PCR | Breast cancer | AQP5 | Expression and |

| | | | | | | |
|---|---|---|---|---|---|---|
| | | In vivo | Small interfering RNA<br>Immunohistochemistry<br>BrdU cell proliferation assay<br>Cell migration assay | | | function |
| [87] | Kang, B. W. 2015 | In vivo | Immunohistochemistry | Colon cancer | AQP1, 3, 5 | Expression |
| [88] | Kang, S. 2015 | In vivo | Immunohistochemistry | Breast cancer | AQP3 | Expression |
| [89] | Kang, S. K. 2008 | Biopsy | Small interfering RNA<br>Immunoblotting<br>Cell proliferation assay | Colon cancer | AQP5 | Expression and function |
| [90] | Kao, S. C. H. 2012 | In vivo | Immunohistochemistry | Pleural Malignant Mesothelioma | AQP1 | Expression |
| [91] | Kasimir-Bauer, S. 2009 | In vivo | Immunohistochemistry | Breast cancer | AQP5 | Expression |
| [92] | Katsurahara, K. 2018 | Biopsy | Western blot<br>Small interfering RNA<br>Apoptosis assay<br>RT PCR<br>Migration assay<br>Invasion assay<br>Immunofluorescence<br>Flow cytometer | Liver cancer | AQP5 | Expression and function |
| [93] | Klebe, S. 2015 | Biopsy<br>In vivo | Small interfering RNA<br>qRT PCR<br>Migration assay<br>Immunohistochemistry<br>Cell proliferation assay<br>Anchorage-Independent Assay | Mesothelioma | AQP1 | Expression and function |
| [94] | Kon, T. 2017 | In vivo | MRI<br>Immunohistochemistry | Esophageal cancer | AQP4 | Expression |
| [95] | Kong, B. 2016 | Biopsy | Western blot<br>Small interfering RNA<br>RT PCR<br>MTT assay | Colon cancer | AQP1 | Expression and function |
| [96] | Kourghi, M. 2016 | Biopsy | Two Electrode Voltage Clamp Recordings<br>Osmotic Swelling Assays<br>Cytotoxicity<br>Cell migration assay | Colon cancer | AQP1 | Function |

| | | | | | | |
|---|---|---|---|---|---|---|
| [97] | Lee, S. J. 2014 | Biopsy | Immunohistochemistry | Breast cancer | AQP5 | Expression |
| [98] | Lee, S. J. 2017 | Biopsy | Haplotype Identification and Reconstruction | Breast cancer | AQP5 | Expression |
| [99] | Lehnerdt, G. F. 2015 | Biopsy | Immunohistochemistry | Pharyngeal squamous cell carcinoma | AQP1<br>AQP5 | Expression |
| [100] | Leung, J. 2007 | In vivo | Western blot<br>qPCR<br>Arsenic cytotoxicity | Leukemia | AQP9 | Expression |
| [101] | Li, A. 2013 | Biopsy<br>In vivo | Migration assay<br>Western blot<br>Immunohistochemistry | Colon cancer | AQP3 | Expression and function |
| [102] | Li, B. 2012 | Biopsy | Immunohistochemistry | Lung cancer | AQP3 | Expression |
| [103] | Li, C. F. 2016 | Biopsy<br>In vitro<br>In vivo | Western Blot<br>RT PCR<br>Immunohistochemistry<br>Colony formation assay<br>Cell cycle analysis<br>Apoptosis assay | Liver cancer | AQP9 | Expression and function |
| [104] | Li, J. 2014 | Biopsy<br>In vivo | Small interfering RNA<br>RT PCR<br>Immunohistochemistry<br>Immunofluorescence assay<br>FISH staining<br>Cell viability<br>Cell invasion assay | Prostate cancer | AQP5 | Expression and function |
| [105] | Li, Q. 2018 | In vivo | Western blot<br>Tumor xenograft study<br>Small interfering RNA<br>RT PCR<br>MTT assay<br>Flow cytometry | Colon cancer | AQP5 | Expression and function |
| [106] | Li, X. 2018 | In vivo | Western blot<br>Small interfering RNA<br>RT PCR<br>MTT assay<br>Flow cytometry | Breast cancer | AQP5 | Expression and function |

| | | | | | | |
|---|---|---|---|---|---|---|
| | | | Cell migration<br>Cell invasion | | | |
| [107] | Li, X. J. 2007 | In vivo | Western blot<br>Immunohistochemistry<br>Carbonic anhydrase activity assay | Lung cancer | AQP1 | Expression and function |
| [108] | Li, Y. B. 2016 | In vitro | Western blot<br>Small interfering RNA<br>RT PCR<br>Migration assay<br>Invasion assay<br>Cell proliferation assay | Breast cancer | AQP4 | Expression and function |
| [109] | Li, Z. 2016 | Biopsy<br>In vitro<br>In vivo | Western blot<br>RT PCR<br>Mouse xenograft model<br>Lipid droplet staining<br>Immunohistochemistry<br>G3P assay<br>Colony formation assay<br>Cellular TAG measurement<br>Cellular glycerol detection<br>Cell proliferation assay<br>ATP assay | Gastric cancer | AQP3 | Expression and function |
| [110] | Liao, Z. Q. 2016 | Biopsy<br>In vitro | Small interfering RNA<br>Western blot<br>RT PCR<br>MTT assay<br>Matrigel invasion assay<br>Dual-Luciferase reporter gene assay<br>ChIP assay<br>Cell migration assays | Brain cancer | AQP1 | Expression and function |
| [111] | Lim, B. C. 2014 | Biopsy | Immunosorbent assay | Brain cancer | AQP4 | Expression |
| [112] | Liu, J. 2015 | Biopsy | Immunohistochemistry | Uroepithelium cancer | AQP1 | Expression |
| [113] | Liu, S. 2013 | Biopsy | Immunohistochemistry | Esophageal cancer | AQP3,5 | Expression |
| [114] | Liu, Y. H. 2015 | In vitro | Western blot<br>RT PCR<br>MTT assay | Lung Cancer | AQP1 | Expression and function |

| | | | | | | |
|---|---|---|---|---|---|---|
| | | | Invasion assay<br>Flow cytometry | | | |
| [115] | Liu, Y. L. 2007 | Biopsy<br>In vitro | Western blot<br>RT PCR<br>Immunohistochemistry | Lung cancer | AQP3 | Expression |
| [116] | Longatti, P. 2006 | Biopsy | Immunohistochemistry | Brain cancer | AQP1 | Expression |
| [117] | Longatti, P. 2006 | Biopsy | Immunohistochemistry | Brain cancer | AQP1 | Expression |
| [118] | Lopez-Campos, J. L. 2011 | Biopsy | Immunohistochemistry | Lung cancer | AQP1 | Expression |
| [119] | Luo, L. 2018 | Biopsy<br>In vitro | qTT-PCR<br>Migration assays<br>Luciferase assays<br>Invasion assays<br>Immunohistochemistry<br>Cell proliferation assays | Breast cancer | AQP1 | Expression and function |
| [120] | Luo, L. M. 2017 | Biopsy | Immunohistochemistry | Liver cancer | AQP1 | Expression |
| [121] | Lv, Y. 2018 | In vitro | Western blot<br>qRT PCR<br>Invasion assay<br>Cell migration assay | Brain cancer | AQP9 | Expression and function |
| [122] | Ma, B. 2004 | In vivo | Western blot<br>Metastases assays<br>Immunohistochemistry<br>Carbonic anhydrase activity assay | Lung cancer | AQP1 | Expression<br>Function |
| [123] | Ma, J. 2016 | Biopsy | Western Blot<br>Immunohistochemistry | Ovarian cancer | AQP6,8 | Expression |
| [124] | Matsuo, K. 2014 | Biopsy | Immunohistochemistry | Oral cancer | AQP3 | Expression |
| [125] | Mazal, P. R. 2005 | Biopsy | Immunohistochemistry | Liver cancer | AQP1 | Expression |
| [126] | McCoy, E. S. 2010 | In vitro<br>In vivo | Western blot<br>Site directed mutagenesis<br>Migration assay<br>Immunocytochemistry<br>Cell volume measurement<br>Cell adhesion assay | Brain cancer | AQP1,4 | Expression and function |
| [127] | Miao, Z. F.2009 | In vitro | MTT cytotoxicity assay<br>Measurement of intracellular glutathione concentration | Lung cancer | AQP9 | Expression and function |

| | | | | | | |
|---|---|---|---|---|---|---|
| | | | Mass spectrometry<br>Immunoblotting<br>Graphite furnace atomic absorption spectrometry<br>Cell proliferation assay | | | |
| [128] | Ming, L. 2008 | Biopsy | Immunohistochemistry | Cervical cancer | AQP1 | Expression |
| [129] | Mobasheri, A. 2005 | Biopsy | Immunohistochemistry | Prostate Cancer<br>Breast Cancer<br>Ovary Cancer<br>Colon cancer<br>Lung cancer | AQP1 | Expression |
| [130] | Mobley, J. 2013 | Biopsy | Western blot | Renal cancer | AQP1 | Expression |
| [131] | Mobley, J. 2013 | Biopsy | Western blot | Renal cancer | AQP1 | Expression |
| [132] | Morrissey, J. J. 2013 | Biopsy | Western blot | Renal cancer | AQP1 | Expression |
| [133] | Morrissey, J. J. 2015 | Biopsy | Western blot | Renal cancer | AQP1 | Expression |
| [134] | Morrissey, J. J. 2014 | Biopsy | Western blot | Renal cancer | AQP1 | Expression |
| [135] | Mou, K. 2010 | Biopsy | Western blot<br>Immunofluorescence<br>Cranial MRI | Brain cancer | AQP4 | Expression |
| [136] | Nakakoshi, M. 2006 | In vitro<br>In vivo | RT PCR<br>Northern blot<br>Immunoblotting | Skin cancer | AQP3 | Expression |
| [137] | Nicchia, G. P. 2013 | In vitro | Western blot<br>Small interfering RNA<br>Proliferation assay<br>Immunofluorescence | Skin cancer | AQP1 | Expression |
| [138] | Niu, D. 2012 | Biopsy<br>In vitro | Western blot<br>RT PCR<br>Immunohistofluorescence<br>Immunohistochemistry | Thyroid cancer | AQP3,4 | Expression |
| [139] | Noell, S. 2015 | Biopsy | RT PCR<br>Electron microscopy<br>Immunohistochemistry | Brain cancer | AQP1,4 | Expression |
| [140] | Noell, S. 2012 | Biopsy<br>In vitro<br>In vivo | Western blot<br>RT PCR<br>Immunohistochemistry<br>Freeze Fracture | Brain cancer | AQP4 | Expression |

| | | | | | | |
|---|---|---|---|---|---|---|
| [141] | Noell, S. 2012 | Biopsy | Immunohistochemistry<br>Electron microscopy<br>Immunogold labeling<br>Immunoblotting<br>Electrophoresis | Brain cancer | AQP4 | Expression |
| [142] | Oshio, K. 2005 | Biopsy | Western blot<br>RT PCR<br>Immunohistochemistry<br>Gene Array | Brain cancer | AQP1 | Expression |
| [143] | Otterbach, F. 2010 | Biopsy | Immunohistochemistry | Breast cancer | AQP1 | Expression |
| [144] | Padma, S. 2009 | Biopsy | Immunohistochemistry<br>Immunofluorescent | Liver cancer | AQP9 | Expression |
| [145] | Pan, H. 2008 | Biopsy | Immunohistochemistry | Skin cancer | AQP1 | Expression |
| [146] | Pan, X. Y. 2012 | In vitro | Western blot<br>Small interfering RNA<br>Migration assay<br>Invasion assay<br>Dual-luciferase reporter assay<br>Cell proliferation assay | Prostate cancer | AQP1 | Expression and function |
| [147] | Park, J. Y. 2017 | Biopsy | Immunohistochemistry | Prostate cancer | AQP1 | Expression |
| [148] | Pei, J. V. 2016 | In vitro | Quantitative Oocyte Swelling Assays<br>qRT PCR<br>Western blot<br>Immunocytochemistry<br>Molecular Docking<br>Migration assay<br>Live cell imaging<br>Electrophysiology<br>Cytotoxicity assay | Colon cancer | AQP1 | Expression and function |
| [149] | Peng, R. 2016 | In vitro | Western blot<br>RT PCR<br>Immunofluorescence<br>Immunocytochemistry<br>Cell proliferation assay | Liver cancer | AQP3,9 | Expression and function |
| [150] | Peng, R. 2016 | Biopsy<br>In vivo | Xenograft tumor model<br>Western blot | Liver cancer | AQP3,9 | Expression |

| | | | | | | |
|---|---|---|---|---|---|---|
| | | | RT PCR<br>ELISA<br>Terminal deoxynucleotidyl transferase-mediated dUTP nick end labeling assay | | | |
| [151] | Prata, C. 2018 | In vitro | Western blot<br>RT PCR<br>Measurement of Intracellular ROS Level<br>Immunofluorescence<br>Electrophoresis<br>Cell Viability | Leukemia | AQP8 | Expression and function |
| [152] | Pulford, E. 2017 | In vitro | Xenograft MM Model<br>VEGFA ELISA<br>Small interfering RNA<br>qRT PCR<br>Matrigel VM assay | Skin cance | AQP1 | Expression and function |
| [153] | Qin, F. 2016 | Biopsy | Western blot<br>Proliferation assay<br>Immunofluorescence<br>Immunocytochemistry<br>Colony formation<br>Matrigel invasion | Breast cancer | AQP1 | Expression and function |
| [154] | Qiu, J. 2018 | Biopsy<br>In vitro | Western blot<br>RT PCR<br>Luciferase reporter assay<br>Invasion Assay<br>Cell proliferation | Bone cancer | AQP3 | Expression and function |
| [155] | Rentsch, C. A. 2009 | Biopsy | qRT PCR | Renal cancer | AQP1 | Expression |
| [156] | Rouzaire-Dubois, B. 2009 | In vitro | RT PCR<br>Small interfering RNA<br>Sodium imaging | Brain cancer | AQP1 | Expression and function |
| [157] | Rubenwolf, P. 2015 | Biopsy | Immunohistochemistry | Bladder cancer | AQP3 | Expression |
| [158] | Rubenwolf, P. C. 2014 | Biopsy<br>In vitro | RT PCR<br>Immunohistochemistry<br>Immunofluorescence | Urothelial cancer | AQP3 | Expression |
| [159] | Saadoun, S. 2002 | Biopsy | Immunohistochemistry | Brain cancer | AQP1 | Expression |

| | | | | | | |
|---|---|---|---|---|---|---|
| [160] | Saadoun, S. 2003 | Biopsy | Immunohistochemistry | Brain cancer | AQP4 | Expression |
| [161] | Saito, Y. 2013 | In vitro<br>In vivo | Western blot<br>Glycerol Uptake assay<br>Immunohistochemistry | Ovarian cancer<br>Brain cancer<br>Breast cancer | AQP3,9 | Expression and function |
| [162] | Sato, K. 2018 | Biopsy | Immunohistochemistry | Ovarian cancer | AQP1,3,5,9 | Expression |
| [163] | Satooka, H. 2016 | In vitro | Time-lapse imaging of chemotaxis and H2O2 uptake.<br>Spontaneous metastasis<br>RT PCR<br>PTP1B oxidation<br>PTEN oxidation<br>Osmotic water permeability assay<br>Migration assay<br>Immunofluorescence<br>Invasion assay<br>Immunoblotting | Breast cancer | AQP3 | Expression and function |
| [164] | Sawada, T. 2007 | Biopsy | Immunohistochemistry | Brain cancer | AQP4 | Expression |
| [165] | Schob, S.2017 | Biopsy | Immunohistochemistry | Brain cancer | AQP4 | Expression |
| [166] | Sekine, S. 2014 | Biopsy | Immunohistochemistry | Biliary tract cancer | AQP1 | Expression |
| [167] | Sekine, S. 2012 | Biopsy | Immunohistochemistry | Biliary tract cancer | AQP5 | Expression |
| [168] | Sekine, S. 2014 | In vitro | Small interfering RNA<br>RT PCR<br>Migration assay<br>Invasion assay<br>Immunohistochemistry<br>Cell proliferation assay | Gallbladder cancer | AQP5 | Expression and function |
| [169] | Seleit, I. 2015 | Biopsy | Immunohistochemistry | Skin cancer | AQP3 | Expression |
| [170] | Shan, T. 2014 | Biopsy | Western blot<br>RT PCR<br>Immunohistochemistry<br>Immunofluorescence<br>FISH staining | Colon cancer | AQP5 | Expression |
| [171] | Shan, T. 2014 | Biopsy | Immunohistochemistry | Colon cancer | AQP5 | Expression |
| [172] | Shen, Q. 2016 | Biopsy | Immunohistochemistry | Cervical cancer | AQP1,3,4,5,8 | Expression |
| [173] | Shi, X. 2013 | In vitro | Western Blot<br>Small interfering RNA | Colon cancer | AQP5 | Expression and function |

| | | | | | | |
|---|---|---|---|---|---|---|
| | | | RT PCR<br>Proliferation assay<br>Flow cytometry | | | |
| [174] | Shi, X. 2014 | Biopsy<br>In vitro | Western blot<br>Sulforhodamine B staining<br>Small interfering RNA<br>qRT PCR<br>Immunohistochemistry | Colon cancer | AQP5 | Expression and function |
| [175] | Shi, X. M. 2015 | In vitro | Western Blot<br>Small interfering RNA<br>MTT assay<br>Flow cytometry | Colon cancer | AQP5 | Expression and function |
| [176] | Shi, Y. H. 2012 | Biopsy | RT PCR<br>Immunohistochemistry<br>Immunofluorescence | Cervical cancer | AQP1,3,8 | Expression |
| [177] | Shi, Y. H. 2013 | In vitro | Xenografts model<br>Migration assay<br>Invasion assay<br>Cell proliferation<br>Adherence assay | Cervical cancer | AQP8 | Function |
| [178] | Shi, Y. H. 2014 | In vitro | Western blot<br>Migration assay<br>Immunofluorescence | Cervical cancer | AQP8 | Expression and function |
| [179] | Shi, Z. 2012 | Biopsy | Western Blot<br>RT PCR<br>Immunohistochemistry | Breast cancer | AQP0-12 | Expression |
| [180] | Shimasaki, M. 2011 | Biopsy | Western Blot<br>qRT PCR<br>Immunostaining | Lung cancer | AQP1,3,5 | Expression |
| [181] | Shimizu, H. 2014 | In vitro<br>Biopsy | Western blot<br>Small interfering RNA<br>RT PCR<br>cell proliferation assay<br>Cell cycle analysis<br>Apoptosis assay | Esophageal cancer | AQP5 | Expression and function |
| [182] | Simone, L. 2018 | In vitro<br>In vivo | Xenograft model<br>Western blot | Skin cancer | AQP1 | Expression and function |

| | | | | | | |
|---|---|---|---|---|---|---|
| | | | Small interfering RNA<br>Immunofluorescence | | | |
| [183] | Smith, E. 2018 | In vitro | Western blot<br>qRT PCR<br>Cell growth assay<br>Cell cycle analysis<br>Apoptosis<br>Acridine Orange Staining | Colon cancer | AQP1 | Expression and function |
| [184] | Smith, E. 2019 | Biopsy<br>In vitro | qRT PCR<br>5-aza-2'-deoxycytidine assay | Colon cancer | AQP1 | Expression and function |
| [185] | Song, T. 2015 | Biopsy | Immunohistochemistry | Lung cancer | AQP5 | Expression |
| [186] | Sreedharan, S. 2014 | Biopsy | Western blot | Renal cancer | AQP1 | Expression |
| [187] | Sung, K. K. 2008 | In vitro | Small interfering RNA<br>Immunohistochemistry<br>Immunoblotting<br>Cell proliferation assay | Colon cancer | AQP5 | Expression and function |
| [188] | Thapa, S. 2018 | Biopsy | Immunohistochemistry | Gastric cancer | AQP1-11 | Expression |
| [189] | Ticozzi-Valerio, D. 2007 | Biopsy | Immunoblotting<br>Electrophoresis | Renal cancer | AQP1 | Expression |
| [190] | Vacca, A. 2001 | Biopsy | Western blot<br>Immunoblotting<br>Bone marrow angiogenesis staining | Skin cancer | AQP1 | Expression |
| [191] | Wang, D. 2011 | Biopsy | Western blot<br>RT PCR<br>Immunohistochemistry | Brain cancer | AQP1,4 | Expression |
| [192] | Warth, A. 2011 | Biopsy | Western blot<br>qRT PCR<br>Immunohistochemistry | Lung cancer | AQP4 | Expression |
| [193] | Warth A. 2007 | Biopsy | Immunohistochemistry | Brain cancer | AQP4 | Expression |
| [194] | Wu, D. Q. 2018 | Biopsy<br>In vitro | Xenograft model<br>Western blot<br>qPCR<br>Migration assay<br>Invasion assay<br>Immunofluorescence<br>Colony formation assay | Colon cancer | AQP8 | Expression and function |

| | | | | | | |
|---|---|---|---|---|---|---|
| | | | Cell proliferation | | | |
| [195] | Xia, H. 2013 | In vitro | Western blot<br>RT PCR<br>MTT<br>Invasion assay<br>Flow cytometry | Lung cancer | AQP1 | Expression and function |
| [196] | Xiong, W. 2018 | Biopsy<br>In vivo | Western blot<br>Small interfering RNA<br>qRT PCR<br>Migration assay<br>Invasion assay<br>Immunohistochemistry<br>Immunofluorescence<br>H&E staining<br>Dual-luciferase reporter assay | Brain cancer | AQP4 | Expression and function |
| [197] | Xuejun, C. 2014 | In vitro | PCR<br>MTT assay | Ovarian cancer | AQP1-10 | Expression and function |
| [198] | Yang, C. 2016 | Biopsy | qRT PCR<br>Immunohistochemistry | Ovarian cancer | AQP3 | Expression |
| [199] | Yang, J. 2012 | In vitro | Western blot<br>RT PCR<br>Growth rate assay | Ovarian cancer | AQP5 | Expression and function |
| [200] | Yang, J. 2017 | Biopsy<br>In vitro | Western blot<br>qRT PCR<br>MTT assay<br>Migration assay<br>Immunohistochemistry<br>Flow cytometry | Brain cancer | AQP5 | Expression and function |
| [201] | Yang, J. H. 2006 | Biopsy | Immunohistochemistry | Ovarian cancer | AQP1 | Expression |
| [202] | Yang, J. H. 2006 | Biopsy | Immunohistochemistry<br>Western blot<br>RT PCR | Ovarian cancer | AQP5 | Expression |
| [203] | Yang, J. H. 2011 | EX-vivo | Western blot<br>Immunohistochemistry | Ovarian cancer | AQP3,7,9 | Expression |
| [204] | Yang, J. H. 2011 | Biopsy | Immunohistochemistry | Ovarian cancer | AQP1-9 | Expression |
| [205] | Yang, L. 2012 | In vitro<br>In vivo | Xenograft glioma model<br>VEGF activity assay | Brain cancer | AQP4 | Expression and function |

| | | | | | | |
|---|---|---|---|---|---|---|
| | | | Tumor vessel permeability assay<br>The water contents of tumor tissue assays<br>qPCR<br>Immunohistochemistry<br>ELISA | | | |
| [206] | Yang, W. C. 2015 | Biopsy | Western blot<br>Immunohistochemistry | Brain cancer | AQP4,9 | Expression |
| [207] | Yang, Z. H. 2015 | In vitro<br>In vivo | Xenograft model<br>Western blot<br>Immunostaining<br>Flow cytometry<br>Cell proliferation assay<br>Cell apoptosis | Colon cancer | AQP9 | Expression and function |
| [208] | Yin, T. 2008 | Biopsy | Immunohistochemistry | Breast cancer | AQP1 | Expression |
| [209] | Yong, J. 2009<br>[209][174][89][121][81][121][81][53][53][53][53][53][210][210][211][211] | In vitro<br>In vivo | Xenograft model<br>Water Permeability Measurements<br>RT PCR<br>Migration assay<br>Measurement of RhoA and Rac Activities<br>Invasion assay<br>Immunofluorescence<br>Immunoblotting | Colon cancer | AQP1 | Expression and function |
| [212] | Yoshida, T. 2013 | Biopsy | Immunostaining<br>H&E staining | Colon cancer | AQP1 | Expression |
| [213] | Yun, S. 2016 | Biopsy | Immunohistochemistry | Lung cancer | AQP1 | Expression |
| [214] | Zhang, L. 2018 | In vitro<br>In vivo | Xenograft model<br>Western blot<br>TUNEL assay<br>qRT PCR<br>MTT assay<br>Flow cytometry<br>Colony formation assay. | Lung cancer | AQP5 | Expression and function |
| [215] | Zhang, L. 2018 | Biopsy | Pet/CT scan<br>Immunohistochemistry | Cervical cancer | AQP1 | Expression |

| | | | | | | |
|---|---|---|---|---|---|---|
| [216] | Zhang, T. 2013 | Biopsy | Western blot<br>qRT PCR<br>Migration assay<br>Luciferase assays<br>Invasion assay<br>Immunohistochemistry<br>Cell proliferation assay | Breast cancer | AQP1 | Expression and function |
| [217] | Zhang, T. 2012 | Biopsy | Western Blot<br>qRT PCR<br>Immunohistochemistry | Cervical cancer | AQP5 | Expression |
| [218] | Zhang, W. G. 2016 | Biopsy<br>In vitro<br>In vivo | Western Blot<br>Small interfering RNA<br>qRT PCR<br>Invasion assay<br>Immunohistochemistry<br>Cell migration assay | Liver cancer | AQP9 | Expression and function |
| [219] | Zhang, X. 2018 | In vitro | Western Blot<br>qRT PCR<br>MTT assay<br>Flow cytometry | Bladder Cancer | AQP1 | Expression and function |
| [220] | Zhang, Z. 2010 | In vitro<br>In vivo | Xenograft model<br>Western blot<br>Small interfering RNA<br>RT PCR<br>Osmotic water permeability measurement<br>Migration assay<br>Invasion assay<br>Immunohistochemistry<br>Immunofluorescence | Lung Cancer | AQP5 | Expression and function |
| [221] | Zhang, Z. 2019 | Biopsy<br>In vivo | Western blot<br>qRT PCR<br>Luciferase reporter assay<br>Flow cytometry assay<br>Detection of HBsAg and HBeAg<br>Cell viability assay<br>Cell proliferation assay | Liver cancer | AQP5 | Expression and function |

| | | | | | | |
|---|---|---|---|---|---|---|
| | | | Caspase-3 activity assay<br>Apoptosis | | | |
| [222] | Zhang, Z. Q. 2011 | In vitro<br>In vivo | Xenograft model<br>Western Blot<br>RT PCR<br>Immunohistochemistry<br>Immunofluorescence | Lung cancer | AQP5 | Expression and function |
| [223] | Zhao, W. J. 2012 | Biopsy<br>In vitro | Immunohistochemistry<br>Immunofluorescence | Brain cancer | AQP4 | Expression |
| [224] | Zhu, S. J. 2013 | Biopsy | Immunohistochemistry<br>Immunoblotting<br>RT PCR | Brain cancer | AQP8 | Expression |
| [225] | Zou, L. B. 2011 | Biopsy<br>In vitro | Western blot<br>siRNA<br>qRT PCR<br>Migration assay<br>Luciferase reporter assay<br>Invasion assay<br>Immunohistochemistry<br>Immunofluorescence<br>Electron microscopic<br>Cell proliferation<br>Adhesion assay | Endometrial cancer | AQP2 | Expression and function |
| [226] | Zou, W. 2019 | Biopsy | Western blot<br>Immunohistochemistry | Pancreatic cancer | AQP1,3 | Expression |

## References


1. Abdelrahman, A.E.; Fathy, A.; Elsebai, E.A.; Nawar, N.; Etman, W.M. Prognostic impact of Apaf-1, Cyclin D1, and AQP-5 in serous ovarian carcinoma treated with the first-line chemotherapy. *Ann. Diagn. Pathol.* **2018**, *35*, 27–37, doi:10.1016/j.anndiagpath.2018.02.005.
2. Allory, Y.; Bazille, C.; Vieillefond, A.; Molinié, V.; Cochand-Priollet, B.; Cussenot, O.; Callard, P.; Sibony, M. Profiling and classification tree applied to renal epithelial tumours. *Histopathol.* **2007**, *52*, 158–166, doi:10.1111/j.1365-2559.2007.02900.x.
3. Angelico, G.; Caltabiano, R.; Loreto, C.; Ieni, A.; Tuccari, G.; Ledda, C.; Rapisarda, V. Immunohistochemical Expression of Aquaporin-1 in Fluoro-Edenite-Induced Malignant Mesothelioma: A Preliminary Report. *Int. J. Mol. Sci.* **2018**, *19*, 685, doi:10.3390/ijms19030685.
4. Angelico, G.; Ieni, A.; Caltabiano, R.; Zeppa, P.; Tuccari, G. Aquaporin-1 expression in fluoro-edenite-induced mesothelioma effusions: An approach by cell-block procedure. *Cytopathol.* **2018**, *29*, 455–460, doi:10.1111/cyt.12583.
5. Aras, Y.; Erguven, M.; Aktas, E.; Yazihan, N.; Bilir, A. Antagonist activity of the antipsychotic drug lithium chloride and the antileukemic drug imatinib mesylate during glioblastoma treatmentin vitro. *Neurol. Res.* **2016**, *38*, 766–774, doi:10.1080/01616412.2016.1203096.
6. Arif, M.; Kitchen, P.; Conner, M.T.; Hill, E.J.; Nagel, D.; Bill, R.M.; Dunmore, S.J.; Armesilla, A.L.; Gross, S.; Carmichael, A.R.; et al. Downregulation of aquaporin 3 inhibits cellular proliferation, migration and invasion in the MDA-MB-231 breast cancer cell line. *Oncol. Lett.* **2018**, *16*, 713–720, doi:10.3892/ol.2018.8759.
7. Bellezza, G.; Vannucci, J.; Bianconi, F.; Metro, G.; Del Sordo, R.; Andolfi, M.; Ferri, I.; Siccu, P.; Ludovini, V.; Puma, F.; et al. Prognostic implication of aquaporin 1 overexpression in resected lung adenocarcinoma†. *Interact. Cardiovasc. Thorac. Surg.* **2017**, *25*, 856–861, doi:10.1093/icvts/ivx202.
8. Ben, Y.; Chen, J.; Zhu, R.; Gao, L.; Bai, C. Upregulation of AQP3 and AQP5 induced by dexamethasone and ambroxol in A549 cells. *Respir. Physiol. Neurobiol.* **2008**, *161*, 111–118, doi:10.1016/j.resp.2007.12.007.
9. Bhattacharjee, H.; Carbrey, J.; Rosen, B.; Mukhopadhyay, R. Drug uptake and pharmacological modulation of drug sensitivity in leukemia by AQP9. *Biochem. Biophys. Res. Commun.* **2004**, *322*, 836–841, doi:10.1016/j.bbrc.2004.08.002.
10. Breyer, J.; Otto, W.; Burger, M.; Hartmann, A.; Rubenwolf, P.C. Aquaporin 3 Expression Loss in Urothelial Carcinoma: Association with Tumor Invasion Depth, but not with Grading? *Bl. Cancer* **2017**, *3*, 31–34, doi:10.3233/blc-160082.
11. Cagini, L.; Balloni, S.; Ludovini, V.; Andolfi, M.; Matricardi, A.; Potenza, R.; Vannucci, J.; Siggillino, A.; Tofanetti, F.R.; Bellezza, G.; et al. Variations in gene expression of lung macromolecules after induction chemotherapy for lung cancer†. *Eur. J. Cardio-Thoracic Surg.* **2017**, *52*, 1077–1082, doi:10.1093/ejcts/ezx200.
12. Cao, X.-C.; Zhang, W.-R.; Cao, W.-F.; Liu, B.-W.; Zhang, F.; Zhao, H.-M.; Meng, R.; Zhang, L.; Niu, R.-F.; Hao, X.-S.; et al. Aquaporin3 Is Required for FGF-2-Induced Migration of Human Breast Cancers. *PLOS ONE* **2013**, *8*, e56735, doi:10.1371/journal.pone.0056735.
13. Capoccia, E.; Cirillo, C.; Marchetto, A.; Tiberi, S.; Sawikr, Y.; Pesce, M.; D'Alessandro, A.; Scuderi, C.; Sarnelli, G.; Cuomo, R.; et al. S100B-p53 disengagement by pentamidine promotes apoptosis and inhibits cellular migration via aquaporin-4 and metalloproteinase-2 inhibition in C6 glioma cells. *Oncol. Lett.* **2015**, *9*, 2864–2870, doi:10.3892/ol.2015.3091.
14. Chae, Y.K.; Kang, S.K.; Kim, M.S.; Woo, J.; Lee, J.; Chang, S.; Kim, D.-W.; Kim, M.; Park, S.; Kim, I.; et al. Human AQP5 Plays a Role in the Progression of Chronic Myelogenous Leukemia (CML). *PLOS ONE* **2008**, *3*, e2594, doi:10.1371/journal.pone.0002594.
15. Chae, Y.K.; Woo, J.; Kim, M.-J.; Kang, S.K.; Kim, M.S.; Lee, J.; Lee, S.K.; Gong, G.; Kim, Y.H.; Soria, J.C.; et al. Expression of Aquaporin 5 (AQP5) Promotes Tumor Invasion in Human Non Small Cell Lung Cancer. *PLOS ONE* **2008**, *3*, e2162, doi:10.1371/journal.pone.0002162.
16. Chae, Y.S.; Lee, S.J.; Lee, J.; Jung, J.H.; Park, H.Y. Abstract P6-01-17: AQP3 expression predicts survival in patients with HER2-positive early breast cancer. *Poster Session Abstracts* **2015**, 75,, doi:10.1158/1538-7445.sabcs14-p6-01-17.
17. Chang, H.; Shi, Y.; Tuokan, T.; Chen, R.; Wang, X. Expression of aquaporin 8 and phosphorylation of Erk1/2 in cervical epithelial carcinogenesis: correlation with clinicopathological parameters. International journal of clinical and experimental pathology 2014, 7, 3928-3937.

18. Chang, H.; Shi, Y.H.; Talaf, T.K.; Lin, C. Aquaporin-8 mediates human esophageal cancer Eca-109 cell migration via the EGFR-Erk1/2 pathway. International journal of clinical and experimental pathology 2014, 7, 7663-7671.
19. Chau, D.; Ng, K.; Chan, T.S.-Y.; Cheng, Y.-Y.; Fong, B.; Tam, S.; Kwong, Y.L.; Tse, E. Azacytidine sensitizes acute myeloid leukemia cells to arsenic trioxide by up-regulating the arsenic transporter aquaglyceroporin 9. *J. Hematol. Oncol.* **2015**, *8*, 1–11, doi:10.1186/s13045-015-0143-3.
20. Chen, C.; Ma, T.; Zhang, C.; Zhang, H.; Bai, L.; Kong, L.; Luo, J. Down-regulation of aquaporin 5-mediated epithelial-mesenchymal transition and anti-metastatic effect by natural product Cairicoside E in colorectal cancer. *Mol. Carcinog.* **2017**, *56*, 2692–2705, doi:10.1002/mc.22712.
21. Chen, G.; Shi, Y.; Liu, M.; Sun, J. circHIPK3 regulates cell proliferation and migration by sponging miR-124 and regulating AQP3 expression in hepatocellular carcinoma. *Cell Death Dis.* **2018**, *9*, 175, doi:10.1038/s41419-017-0204-3.
22. Chen, J.; Bai, C.; Zhang, M.; Ren, Z.; Hu, J. [Expression of aquaporins and its significance in human pulmonary adenocarcinoma cell line SPC-A-1]. *Zhongguo Fei Ai Za Zhi* 2004, *7*, 199–201.
23. Chen, J.; Wang, T.; Zhou, Y.-C.; Gao, F.; Zhang, Z.-H.; Xu, H.; Wang, S.-L.; Shen, L. Aquaporin 3 promotes epithelial-mesenchymal transition in gastric cancer. *J. Exp. Clin. Cancer Res.* **2014**, *33*, 38, doi:10.1186/1756-9966-33-38.
24. Chen, J.; Wang, Z.; Xu, D.; Liu, Y.; Gao, Y. Aquaporin 3 promotes prostate cancer cell motility and invasion via extracellular signal-regulated kinase 1/2-mediated matrix metalloproteinase-3 secretion. *Mol. Med. Rep.* **2014**, *11*, 2882–2888, doi:10.3892/mmr.2014.3097.
25. Chen, L.; Li, Z.; Zhang, Q.; Wei, S.; Li, B.; Zhang, X.; Zhang, L.; Li, Q.; Xu, H.; Xu, Z. Silencing of AQP3 induces apoptosis of gastric cancer cells via downregulation of glycerol intake and downstream inhibition of lipogenesis and autophagy. *OncoTargets Ther.* **2017**, *10*, 2791–2804, doi:10.2147/OTT.S134016.
26. Chen, Q.; Zhu, L.; Zheng, B.; Wang, J.; Song, X.; Zheng, W.; Wang, L.; Yang, D.; Wang, J. Effect of AQP9 Expression in Androgen-Independent Prostate Cancer Cell PC3. *Int. J. Mol. Sci.* **2016**, *17*, 738, doi:10.3390/ijms17050738.
27. Chen, Q.; Zhu, L.; Zong, H.; Song, X.; Wang, L.; Wang, X.; Yang, D.; Wang, J. Subcellular localization of aquaporin 3 in prostate cancer is regulated by RalA. *Oncol. Rep.* **2018**, *39*, 2171–2177, doi:10.3892/or.2018.6308.
28. Chen, R.; Lin, C.; Gulijiahan, A.; Lalai, S. Expression and significance of aquaporin 3(AQP 3) in cervical carcinogenesis. Chinese Journal of Clinical Oncology 2012, 39, 145-148, doi:10.3969/j.issn.1000-8179.2012.03.006.
29. Chen, R.; Shi, Y.; Amiduo, R.; Tuokan, T.; Suzuk, L. Expression and Prognostic Value of Aquaporin 1, 3 in Cervical Carcinoma in Women of Uygur Ethnicity from Xinjiang, China. *PLOS ONE* **2014**, *9*, e98576, doi:10.1371/journal.pone.0098576.
30. Xuejun, C.; Weimin, C.; Xiaoyan, D.; Wei, Z.; Qiong, Z.; Jianhua, Y. Effects of aquaporins on chemosensitivity to cisplatin in ovarian cancer cells. *Arch. Gynecol. Obstet.* **2014**, *290*, 525–532, doi:10.1007/s00404-014-3216-6.
31. Chen, X.; Zhou, C.; Yan, C.; Ma, J.; Zheng, W. Hyperosmotic stress induces cisplatin sensitivity in ovarian cancer cells by stimulating aquaporin-5 expression. *Exp. Ther. Med.* **2015**, *10*, 2055–2062, doi:10.3892/etm.2015.2833.
32. Chen, X.-F.; Li, C.-F.; Lu, L.; Mei, Z.-C. Expression and clinical significance of aquaglyceroporins in human hepatocellular carcinoma. *Mol. Med. Rep.* **2016**, *13*, 5283–5289, doi:10.3892/mmr.2016.5184.
33. Chen, Y.; Gao, F.; Jiang, R.; Liu, H.; Hou, J.; Yi, Y.; Kang, L.; Liu, X.; Li, Y.; Yang, M. Down-Regulation of AQP4 Expression via p38 MAPK Signaling in Temozolomide-Induced Glioma Cells Growth Inhibition and Invasion Impairment. *J. Cell. Biochem.* **2017**, *118*, 4905–4913, doi:10.1002/jcb.26176.
34. Chen, Y.; Miller, C.; Mosher, R.; Zhao, X.; Deeds, J.; Morrissey, M.; Bryant, B.; Yang, D.; Meyer, R.; Cronin, F.; et al. Identification of cervical cancer markers by cDNA and tissue microarrays. *Cancer Res.* 2003, *63*, 1927–1935.
35. Chen, Y.; Tachibana, O.; Oda, M.; Xu, R.; Hamada, J.-I.; Yamashita, J.; Hashimoto, N.; Takahashi, J.A. Increased expression of aquaporin 1 in human hemangioblastomas and its correlation with cyst formation. *J. Neuro-Oncology* **2006**, *80*, 219–225, doi:10.1007/s11060-005-9057-1.
36. Chen, Z.; Zhang, Z.; Gu, Y.; Bai, C. Impaired migration and cell volume regulation in aquaporin 5-deficient SPC-A1 cells. *Respir. Physiol. Neurobiol.* **2011**, *176*, 110–117, doi:10.1016/j.resp.2011.02.001.

37. Deb, P.; Pal, S.; Dutta, V.; Boruah, D.; Chandran, V.; Bhatoe, H.; P, D.; S, P.; V, D.; D, B.; et al. Correlation of expression pattern of aquaporin-1 in primary central nervous system tumors with tumor type, grade, proliferation, microvessel density, contrast-enhancement and perilesional edema. *J. Cancer Res. Ther.* **2012**, *8*, 571, doi:10.4103/0973-1482.106542.
38. Gu, F.; Ding, T.; Ma, Y.; Li, W.; Liu, X.; Ying, G.; Fu, L. Role of aquaporin-4 in the regulation of migration and invasion of human glioma cells. *Int. J. Oncol.* **2011**, *38*, 1521–1531, doi:10.3892/ijo.2011.983.
39. Ding, T.; Zhou, Y.; Sun, K.; Jiang, W.; Li, W.; Liu, X.; Tian, C.; Li, Z.; Ying, G.; Fu, L.; et al. Knockdown a Water Channel Protein, Aquaporin-4, Induced Glioblastoma Cell Apoptosis. *PLOS ONE* **2013**, *8*, e66751, doi:10.1371/journal.pone.0066751.
40. Dong, X.; Wang, Y.; Zhou, Y.; Wen, J.; Wang, S.; Shen, L. Aquaporin 3 facilitates chemoresistance in gastric cancer cells to cisplatin via autophagy. *Cell Death Discov.* **2016**, *2*, 16087, doi:10.1038/cddiscovery.2016.87.
41. Dorward, H.S.; Du, A.; Bruhn, M.A.; Wrin, J.; Pei, J.V.; Evdokiou, A.; Price, T.J.; Yool, A.J.; Hardingham, J.E. Pharmacological blockade of aquaporin-1 water channel by AqB013 restricts migration and invasiveness of colon cancer cells and prevents endothelial tube formation in vitro. *J. Exp. Clin. Cancer Res.* **2016**, *35*, 36, doi:10.1186/s13046-016-0310-6.
42. Dou, R.; Deng, Y.; Huang, L.; Fu, S.; Tan, S.; Wang, L.; Lian, L.; Fang, L.; Fan, X.; Jin, G.; et al. Multi-microarray identifies lower AQP9 expression in adjuvant chemotherapy nonresponders with stage III colorectal cancer. *Cancer Lett.* **2013**, *336*, 106–113, doi:10.1016/j.canlet.2013.04.017.
43. INVITED ABSTRACTS. *J. Thorac. Oncol.* **2013**, *8*, S2–S1348, doi:10.1097/01.jto.0000438438.14562.c8.
44. Driml, J.; Pulford, E.; Moffat, D.; Karapetis, C.; Kao, S.; Griggs, K.; Henderson, D.; Klebe, S. Usefulness of Aquaporin 1 as a Prognostic Marker in a Prospective Cohort of Malignant Mesotheliomas. *Int. J. Mol. Sci.* **2016**, *17*, 1041, doi:10.3390/ijms17071041.
45. Dua, R.K.; Devi, B.I.; Yasha, T.C. Increased expression of Aquaporin-4 and its correlation with contrast enhancement and perilesional edema in brain tumors. *Br. J. Neurosurg.* **2010**, *24*, 454–459, doi:10.3109/02688691003739873.
46. El Hindy, N.; Bankfalvi, A.; Herring, A.; Adamzik, M.; Lambertz, N.; Zhu, Y.; Siffert, W.; Sure, U.; Sandalcioglu, I.E. Correlation of aquaporin-1 water channel protein expression with tumor angiogenesis in human astrocytoma. *Anticancer. Res.* 2013, *33*, 609–613.
47. El Hindy, N.; Rump, K.; Lambertz, N.; Zhu, Y.; Frey, U.H.; Bankfalvi, A.; Siffert, W.; Sure, U.; Peters, J.; Adamzik, M.; et al. The functional Aquaporin 1 −783G/C-polymorphism is associated with survival in patients with glioblastoma multiforme. *J. Surg. Oncol.* **2013**, *108*, 492–498, doi:10.1002/jso.23421.
48. Endo, M.; Jain, R.K.; Witwer, B.; Brown, D. Water Channel (Aquaporin 1) Expression and Distribution in Mammary Carcinomas and Glioblastomas. *Microvasc. Res.* **1999**, *58*, 89–98, doi:10.1006/mvre.1999.2158.
49. Esghaei, M.; Ghaffari, H.; Esboei, B.R.; Tapeh, Z.E.; Salim, F.B.; Motevalian, M. Evaluation of Anticancer Activity of Camellia Sinensis in the Caco-2 Colorectal Cancer Cell Line. *Asian Pac. J. Cancer Prev. 19*, 1697–1701.
50. Evans, J.; Akhter, A.; Carbone, D.; Dikov, M.; Tchekneva, E. P2.03b-082 AQP11 as a Novel Factor of Lung Cancer Cell Resistance to Cisplatin. *J. Thorac. Oncol.* **2017**, *12*, S985–S986, doi:10.1016/j.jtho.2016.11.1364.
51. Roth, P.; Silginer, M.; Goodman, S.L.; Hasenbach, K.; Thies, S.; Schraml, P.; Tabatabai, G.; Moch, H.; Tritschler, I.; Weller, M.; et al. Abstracts. *Neuro-Oncology* **2012**, *14*, iii1–iii94, doi:10.1093/neuonc/nos183.
52. Posters. *Glia* **2013**, *61*, S49–S216, doi:10.1002/glia.22530.
53. Fossdal, G.; Vik-Mo, E.O.; Sandberg, C.; Varghese, M.; Kaarbø, M.; Telmo, E.; Langmoen, I.A.; Murrell, W. Aqp 9 and Brain Tumour Stem Cells. *Sci. World J.* **2012**, *2012*, 1–9, doi:10.1100/2012/915176.
54. Hu, Y.-L.; De Lay, M.; Rose, S.D.; Carbonell, W.S.; Aghi, M.K.; Paquette, J.; Tokuyasu, T.; Tsao, S.; Chaumeil, M.; Ronen, S.; et al. ANGIOGENESIS AND INVASION. *Neuro-Oncology* **2011**, *13*, iii1–iii9, doi:10.1093/neuonc/nor147.
55. Guan, Y.; Chen, J.; Zhan, Y.; Lu, H. Effects of dexamethasone on C6 cell proliferation, migration and invasion through the upregulation of AQP1. *Oncol. Lett.* **2018**, *15*, 7595–7602, doi:10.3892/ol.2018.8269.
56. Guo, K.; Jin, F. NFAT5 promotes proliferation and migration of lung adenocarcinoma cells in part through regulating AQP5 expression. *Biochem. Biophys. Res. Commun.* **2015**, *465*, 644–649, doi:10.1016/j.bbrc.2015.08.078.

57. Guo, X.; Sun, T.; Yang, M.; Li, Z.; Li, Z.; Gao, Y. Prognostic Value of Combined Aquaporin 3 and Aquaporin 5 Overexpression in Hepatocellular Carcinoma. *BioMed Res. Int.* **2013**, *2013*, 1–7, doi:10.1155/2013/206525.
58. Hara-Chikuma, M.; Verkman, A.S. Prevention of Skin Tumorigenesis and Impairment of Epidermal Cell Proliferation by Targeted Aquaporin-3 Gene Disruption. *Mol. Cell. Boil.* **2007**, *28*, 326–332, doi:10.1128/mcb.01482-07.
59. Hara-Chikuma, M.; Watanabe, S.; Satooka, H. Involvement of aquaporin-3 in epidermal growth factor receptor signaling via hydrogen peroxide transport in cancer cells. *Biochem. Biophys. Res. Commun.* **2016**, *471*, 603–609, doi:10.1016/j.bbrc.2016.02.010.
60. Hayashi, Y.; Edwards, N.A.; Proescholdt, M.A.; Oldfield, E.H.; Merrill, M.J. Regulation and Function of Aquaporin-1 in Glioma Cells. *Neoplasia* **2007**, *9*, 777–787, doi:10.1593/neo.07454.
61. He, Z.; Dong, W.; Hu, J.; Ren, X. AQP5 promotes hepatocellular carcinoma metastasis via NF-κB-regulated epithelial-mesenchymal transition. *Biochem. Biophys. Res. Commun.* **2017**, *490*, 343–348, doi:10.1016/j.bbrc.2017.06.046.
62. El Hindy, N.; Bankfalvi, A.; Herring, A.; Adamzik, M.; Lambertz, N.; Zhu, Y.; Siffert, W.; Sure, U.; Sandalcioglu, I.E. Correlation of aquaporin-1 water channel protein expression with tumor angiogenesis in human astrocytoma. *Anticancer. Res.* 2013, *33*, 609–614.
63. Hoque, M.O.; Soria, J.-C.; Woo, J.; Lee, T.; Lee, J.; Jang, S.J.; Upadhyay, S.; Trink, B.; Monitto, C.; Desmaze, C.; et al. Aquaporin 1 Is Overexpressed in Lung Cancer and Stimulates NIH-3T3 Cell Proliferation and Anchorage-Independent Growth. *Am. J. Pathol.* **2006**, *168*, 1345–1353, doi:10.2353/ajpath.2006.050596.
64. Hu, H.; Yao, H.-T.; Zhang, W.-P.; Zhang, L.; Ding, W.; Zhang, S.-H.; Chen, Z.; Wei, E.-Q. Increased expression of aquaporin-4 in human traumatic brain injury and brain tumors*. *J. Zhejiang Univ. Sci. B* **2004**, *6*, 33–37, doi:10.1631/jzus.2005.B0033.
65. Huang, D.; Feng, X.; Liu, Y.; Deng, Y.; Chen, H.; Chen, D.; Fang, L.; Cai, Y.; Liu, H.; Wang, L.; et al. AQP9-induced cell cycle arrest is associated with RAS activation and improves chemotherapy treatment efficacy in colorectal cancer. *Cell Death Dis.* **2017**, *8*, e2894, doi:10.1038/cddis.2017.282.
66. Huang, X.; Huang, L.; Shao, M. Aquaporin 3 facilitates tumor growth in pancreatic cancer by modulating mTOR signaling. *Biochem. Biophys. Res. Commun.* **2017**, *486*, 1097–1102, doi:10.1016/j.bbrc.2017.03.168.
67. Huang, Y.; Murakami, T.; Sano, F.; Kondo, K.; Nakaigawa, N.; Kishida, T.; Kubota, Y.; Nagashima, Y.; Yao, M. Expression of Aquaporin 1 in Primary Renal Tumors: A Prognostic Indicator for Clear-Cell Renal Cell Carcinoma. *Eur. Urol.* **2009**, *56*, 690–699, doi:10.1016/j.eururo.2008.10.014.
68. Huang, Y.; Zhu, Z.; Sun, M.; Wang, J.; Guo, R.; Shen, L.; Wu, W. Critical role of aquaporin-3 in the human epidermal growth factor-induced migration and proliferation in the human gastric adenocarcinoma cells. *Cancer Boil. Ther.* **2010**, *9*, 1000–1007, doi:10.4161/cbt.9.12.11705.
69. Huang, Y.-H.; Zhou, X.-Y.; Wang, H.-M.; Xu, H.; Chen, J.; Lv, N.-H. Aquaporin 5 promotes the proliferation and migration of human gastric carcinoma cells. *Tumor Boil.* **2013**, *34*, 1743–1751, doi:10.1007/s13277-013-0712-4.
70. Huang, Y.-T.; Zhou, J.; Shi, S.; Xu, H.-Y.; Qu, F.; Zhang, D.; Chen, Y.-D.; Yang, J.; Huang, H.-F.; Sheng, J.-Z. Identification of Estrogen Response Element in Aquaporin-3 Gene that Mediates Estrogen-induced Cell Migration and Invasion in Estrogen Receptor-positive Breast Cancer. *Sci. Rep.* **2015**, *5*, 12484, doi:10.1038/srep12484.
71. Hwang, I.; Jung, S.-I.; Hwang, E.C.; Song, S.H.; Lee, H.-S.; Kim, S.-O.; Kang, T.-W.; Kwon, D.; Park, K. Expression and Localization of Aquaporins in Benign Prostate Hyperplasia and Prostate Cancer. *Chonnam Med J.* **2012**, *48*, 174–178, doi:10.4068/cmj.2012.48.3.174.
72. Imaizumi, H.; Ishibashi, K.; Takenoshita, S.; Ishida, H. Aquaporin 1 expression is associated with response to adjuvant chemotherapy in stageï¿½II and III colorectal cancer. *Oncol. Lett.* **2018**, *15*, 6450–6456, doi:10.3892/ol.2018.8170.
73. Imrédi, E.; Liszkay, G.; Kenessey, I.; Plotár, V.; Gödény, M.; Tóth, B.; Fedorcsák, I.; Tímár, J. Aquaporin-1 Protein Expression of the Primary Tumor May Predict Cerebral Progression of Cutaneous Melanoma. *Pathol. Oncol. Res.* **2018**, *26*, 405–410, doi:10.1007/s12253-018-0513-6.
74. Imrédi, E.; Tóth, B.; Doma, V.; Barbai, T.; Raso, E.; Kenessey, I.; Timar, J. Aquaporin 1 protein expression is associated with BRAF V600 mutation and adverse prognosis in cutaneous melanoma. *Melanoma Res.* **2016**, *26*, 1–260, doi:10.1097/cmr.0000000000000243.

75. Iriyama, N.; Yuan, B.; Yoshino, Y.; Hatta, Y.; Horikoshi, A.; Aizawa, S.; Takeuchi, J.; Toyoda, H. Aquaporin 9, a promising predictor for the cytocidal effects of arsenic trioxide in acute promyelocytic leukemia cell lines and primary blasts. *Oncol. Rep.* **2013**, *29*, 2362–2368, doi:10.3892/or.2013.2388.
76. Ishimoto, S.; Wada, K.; Usami, Y.; Tanaka, N.; Aikawa, T.; Okura, M.; Nakajima, A.; Kogo, M.; Kamisaki, Y. Differential expression of aquaporin 5 and aquaporin 3 in squamous cell carcinoma and adenoid cystic carcinoma. *Int. J. Oncol.* **2012**, *41*, 67–75, doi:10.3892/ijo.2012.1445.
77. Ismail, M.; Bokaee, S.; Davies, J.; Harrington, K.J.; Pandha, H. Inhibition of the aquaporin 3 water channel increases the sensitivity of prostate cancer cells to cryotherapy. *Br. J. Cancer* **2009**, *100*, 1889–1895, doi:10.1038/sj.bjc.6605093.
78. Isokpehi, R.D.; Valero, K.C.W.; Graham, B.E.; Pacurari, M.; Sims, J.N.; Udensi, U.K.; Ndebele, K. Secondary Data Analytics of Aquaporin Expression Levels in Glioblastoma Stem-Like Cells. *Cancer Informatics* **2015**, *14*, 95–103, doi:10.4137/CIN.S22058.
79. Jagirdar, R.; Solenov, E.; Hatzoglou, C.; Molyvdas, P.-A.; Gourgoulianis, K.I.; Zarogiannis, S.G. Gene expression profile of aquaporin 1 and associated interactors in malignant pleural mesothelioma. *Gene* **2013**, *517*, 99–105, doi:10.1016/j.gene.2012.12.075.
80. Jagirdar, R.M.; Apostolidou, E.; Molyvdas, P.A.; Gourgoulianis, K.I.; Hatzoglou, C.; Zarogiannis, S.G. Influence of AQP1 on cell adhesion, migration, and tumor sphere formation in malignant pleural mesothelioma is substratum- and histological-type dependent. *Am. J. Physiol. Cell. Mol. Physiol.* **2016**, *310*, L489–L495, doi:10.1152/ajplung.00410.2015.
81. Jeleń, S.; Ulhøi, B.P.; Larsen, A.; Frøkiær, J.; Nielsen, S.; Rützler, M. AQP9 Expression in Glioblastoma Multiforme Tumors Is Limited to a Small Population of Astrocytic Cells and CD15+/CalB+ Leukocytes. *PLOS ONE* **2013**, *8*, e75764, doi:10.1371/journal.pone.0075764.
82. Ji, C.; Cao, C.; Lu, S.; Kivlin, R.; Amaral, A.; Kouttab, N.; Yang, H.; Chu, W.; Bi, Z.; Di, W.; et al. Curcumin attenuates EGF-induced AQP3 up-regulation and cell migration in human ovarian cancer cells. *Cancer Chemother. Pharmacol.* **2008**, *62*, 857–865, doi:10.1007/s00280-007-0674-6.
83. Jia, B.; Li, H.; Cha, N.; Bao, W.; Zhao, R.; Sun, S. Inhibition of aquaporin 5 suppresses proliferation, migration, and invasion of breast cancer cells by modulating mapk signaling. Acta Medica Mediterranea 2018, 34, 1397-1403, doi:10.19193/0393-6384_2018_5_213.
84. Jiang, B.; Li, Z.; Zhang, W.; Wang, H.; Zhi, X.; Feng, J.; Chen, Z.; Zhu, Y.; Yang, L.; Xu, H.; et al. miR-874 Inhibits cell proliferation, migration and invasion through targeting aquaporin-3 in gastric cancer. *J. Gastroenterol.* **2013**, *49*, 1011–1025, doi:10.1007/s00535-013-0851-9.
85. Jo, Y.M.; Park, T.I.; Lee, H.Y.; Jeong, J.Y.; Lee, W.K. Prognostic Significance of Aquaporin 5 Expression in Non-small Cell Lung Cancer. *J. Pathol. Transl. Med.* **2016**, *50*, 122–128, doi:10.4132/jptm.2015.10.31.
86. Jung, H.J.; Park, J.-Y.; Jeon, H.-S.; Kwon, T.-H. Aquaporin-5: A Marker Protein for Proliferation and Migration of Human Breast Cancer Cells. *PLOS ONE* **2011**, *6*, e28492, doi:10.1371/journal.pone.0028492.
87. Kang, B.W.; Kim, J.G.; Lee, S.J.; Chae, Y.S.; Jeong, J.Y.; Yoon, G.S.; Park, S.Y.; Kim, H.J.; Park, J.S.; Choi, G.-S. Expression of Aquaporin-1, Aquaporin-3, and Aquaporin-5 Correlates with Nodal Metastasis in Colon Cancer. *Oncol.* **2015**, *88*, 369–376, doi:10.1159/000369073.
88. Kang, S.; Chae, Y.S.; Lee, S.J.; Kang, B.W.; Kim, J.G.; Kim, W.W.; Jung, J.H.; Park, H.Y.; Jeong, J.-H.; Jeong, J.Y.; et al. Aquaporin 3 Expression Predicts Survival in Patients with HER2-positive Early Breast Cancer. *Anticancer. Res.* 2015, *35*, 2775–2782.
89. Kang, S.K.; Chae, Y.K.; Woo, J.; Kim, M.S.; Park, J.C.; Lee, J.; Soria, J.C.; Jang, S.J.; Sidransky, D.; Moon, C. Role of Human Aquaporin 5 In Colorectal Carcinogenesis. *Am. J. Pathol.* **2008**, *173*, 518–525, doi:10.2353/ajpath.2008.071198.
90. Kao, S.C.-H.; Armstrong, N.; Condon, B.; Griggs, K.; McCaughan, B.; Maltby, S.; Wilson, A.; Henderson, D.W.; Klebe, S. Aquaporin 1 is an independent prognostic factor in pleural malignant mesothelioma. *Cancer* **2011**, *118*, 2952–2961, doi:10.1002/cncr.26497.
91. Kasimir-Bauer, S.; Heubner, M.; Otterbach, F.; Kimmig, R.; Siffert, W.; Adamzik, M. Prognostic relevance of the AQP5 ?1364C>A polymorphism in primary breast cancer. *Mol. Med. Rep.* **2009**, *2*, 645–650,, doi:10.3892/mmr_00000151.
92. "Abstracts of the 77th Annual Meeting of the Japanese Cancer Association; 2018 Sept 27-29; Osaka, Japan" as Cancer Science, Supplement 2, Vol 109 (2018). *Cancer Sci.* **2018**, *109*, 1–1444, doi:10.1111/cas.13904.

93. Klebe, S.; Griggs, K.; Cheng, Y.; Driml, J.; Henderson, D.W.; Reid, G. Blockade of Aquaporin 1 Inhibits Proliferation, Motility, and Metastatic Potential of Mesothelioma In Vitro but not in an In Vivo Model. *Dis. Markers* **2015**, *2015*, 1–9, doi:10.1155/2015/286719.
94. Kon, T.; Ueno, T.; Suzuki, C.; Nunomura, J.; Igarashi, S.; Sato, T.; Tomiyama, M. Aquaporin-4 antibody positive neuromyelitis optica spectrum disorder associated with esophageal cancer. *J. Neuroimmunol.* **2017**, *309*, 38–40, doi:10.1016/j.jneuroim.2017.05.009.
95. Kong, B.; Zhao, S.P. Inhibitory effects of lentivirus mediated RNA interference targeting human AQP1 gene on the proliferation of human colon carcinoma SW480 cells and the expression of VEGF. International Journal of Clinical and Experimental Medicine 2016, 9, 8999-9006.
96. Kourghi, M.; Pei, J.V.; De Ieso, M.L.; Flynn, G.; Yool, A.J. Bumetanide Derivatives AqB007 and AqB011 Selectively Block the Aquaporin-1 Ion Channel Conductance and Slow Cancer Cell Migration. *Mol. Pharmacol.* **2015**, *89*, 133–140, doi:10.1124/mol.115.101618.
97. Lee, S.J.; Chae, Y.S.; Kim, J.G.; Kim, W.W.; Jung, J.H.; Park, H.Y.; Jeong, J.Y.; Park, J.-Y.; Jung, H.J.; Kwon, T.-H. AQP5 Expression Predicts Survival in Patients with Early Breast Cancer. *Ann. Surg. Oncol.* **2013**, *21*, 375–383, doi:10.1245/s10434-013-3317-7.
98. Lee, S.J.; Kang, B.W.; Kim, J.G.; Jung, J.H.; Lee, J.; Kim, W.W.; Park, H.Y.; Jeong, J.-H.; Jeong, J.Y.; Park, J.-Y.; et al. AQP5 Variants Affect Tumoral Expression of AQP5 and Survival in Patients with Early Breast Cancer. *Oncol.* **2016**, *92*, 153–160, doi:10.1159/000452715.
99. Lehnerdt, G.F.; Bachmann, H.S.; Adamzik, M.; Panic, A.; Köksal, E.; Weller, P.; Lang, S.; Schmid, K.W.; Siffert, W.; Bankfalvi, A. AQP1, AQP5, Bcl-2 and p16 in pharyngeal squamous cell carcinoma. *J. Laryngol. Otol.* **2015**, *129*, 580–586, doi:10.1017/s002221511500119x.
100. Leung, J.; Pang, A.; Yuen, W.-H.; Kwong, Y.L.; Tse, E. Relationship of expression of aquaglyceroporin 9 with arsenic uptake and sensitivity in leukemia cells. *Blood* **2006**, *109*, 740–746, doi:10.1182/blood-2006-04-019588.
101. Li, A.; Lu, D.; Zhang, Y.; Li, J.; Fang, Y.; Li, F.; Sun, J. Critical role of aquaporin-3 in epidermal growth factor-induced migration of colorectal carcinoma cells and its clinical significance. *Oncol. Rep.* **2012**, *29*, 535–540, doi:10.3892/or.2012.2144.
102. Li, B.; Jin, L.; Zhong, K.; Du, D. Correlation of aquaporin 3 expression with the clinicopathologic characteristics of non-small cell lung cancer. *Chinese Journal of Lung Cancer* **2012**, *15*, 404-408, doi:10.3779/j.issn.1009-3419.2012.07.03.
103. Li, C.-F.; Zhang, W.-G.; Liu, M.; Qiu, L.-W.; Chen, X.-F.; Lv, L.; Mei, Z.-C. Aquaporin 9 inhibits hepatocellular carcinoma through up-regulating FOXO1 expression. *Oncotarget* **2016**, *7*, 44161–44170, doi:10.18632/oncotarget.10143.
104. Li, J.; Wang, Z.; Chong, T.; Chen, H.; Li, H.; Li, G.; Zhai, X.; Li, Y. Over-expression of a poor prognostic marker in prostate cancer: AQP5 promotes cells growth and local invasion. *World J. Surg. Oncol.* **2014**, *12*, 284, doi:10.1186/1477-7819-12-284.
105. Li, Q.; Yang, T.; Li, D.; Ding, F.; Bai, G.; Wang, W.; Sun, H. Knockdown of aquaporin-5 sensitizes colorectal cancer cells to 5-fluorouracil via inhibition of the Wnt–β-catenin signaling pathway. *Biochem. Cell Boil.* **2018**, *96*, 572–579, doi:10.1139/bcb-2017-0162.
106. Li, X.; Pei, B.; Wang, H.; Tang, C.; Zhu, W.; Jin, F. Effect of AQP-5 silencing by siRNA interference on chemosensitivity of breast cancer cells. *OncoTargets Ther.* **2018**, *11*, 3359–3368, doi:10.2147/OTT.S160313.
107. Li, X.-J.; Xiang, Y.; Ma, B.; Qi, X.-Q. Effects of Acetazolamide Combined with or without NaHCO3 on Suppressing Neoplasm Growth, Metastasis and Aquaporin-1 (AQP1) Protein Expression. *Int. J. Mol. Sci.* **2007**, *8*, 229–240, doi:10.3390/i8030229.
108. Li, Y.-B.; Sun, S.-R.; Han, X.-H. Down-regulation of AQP4 Inhibits Proliferation, Migration and Invasion of Human Breast Cancer Cells. *Folia Boil.* 2016, *62*, 131–137.
109. Li, Z.; Li, B.; Zhang, L.; Chen, L.; Sun, G.; Zhang, Q.; Wang, J.; Zhi, X.; Wang, L.; Xu, Z.; et al. The proliferation impairment induced by AQP3 deficiency is the result of glycerol uptake and metabolism inhibition in gastric cancer cells. *Tumor Boil.* **2016**, *37*, 9169–9179, doi:10.1007/s13277-015-4753-8.
110. Liao, Z.-Q.; Ye, M.; Yu, P.-G.; Xiao, C.; Lin, F.-Y. Glioma-Associated Oncogene Homolog1 (Gli1)-Aquaporin1 pathway promotes glioma cell metastasis. *BMB Rep.* **2016**, *49*, 394–399, doi:10.5483/bmbrep.2016.49.7.011.

111. Lim, B.C.; Chae, J.H.; Kim, S.-K.; Park, S.-H.; Wang, K.-C.; Lee, J.Y.; Phi, J.H. Aquaporin-4 autoimmunity masquerading as a brainstem tumor. *J. Neurosurgery: Pediatr.* **2014**, *14*, 301–305, doi:10.3171/2014.6.peds13674.
112. Liu, J.; Zhang, W.; Ding, D.-G. Expression of Aquaporin 1 in Bladder Uroepithelial Cell Carcinoma and its Relevance to Recurrence. *Asian Pac. J. Cancer Prev.* **2015**, *16*, 3973–3976, doi:10.7314/apjcp.2015.16.9.3973.
113. Liu, S.; Zhang, S.-Y.; Jiang, H.; Yang, Y.; Jiang, Y. Co-expression of AQP3 and AQP5 in esophageal squamous cell carcinoma correlates with aggressive tumor progression and poor prognosis. *Med Oncol.* **2013**, *30*, 30,, doi:10.1007/s12032-013-0636-2.
114. Liu, Y.; Zhu, W. Effects of cetuximab combined with afatinib on the expression of KDR and AQP1 in lung cancer. *Genet. Mol. Res.* **2015**, *14*, 16652–16661, doi:10.4238/2015.december.11.12.
115. Liu, Y.L.; Matsuzaki, T.; Nakazawa, T.; Murata, S.-I.; Nakamura, N.; Kondo, T.; Iwashina, M.; Mochizuki, K.; Yamane, T.; Takata, K.; et al. Expression of aquaporin 3 (AQP3) in normal and neoplastic lung tissues. *Hum. Pathol.* **2007**, *38*, 171–178, doi:10.1016/j.humpath.2006.07.015.
116. Longatti, P.; Basaldella, L.; Orvieto, E.; Tos, A.P.D.; Martinuzzi, A. Aquaporin(s) Expression in Choroid Plexus Tumours. *Pediatr. Neurosurg.* **2006**, *42*, 228–233, doi:10.1159/000092359.
117. Longatti, P.; Basaldella, L.; Orvieto, E.; Tos, A.P.D.; Martinuzzi, A. Aquaporin 1 expression in cystic hemangioblastomas. *Neurosci. Lett.* **2006**, *392*, 178–180, doi:10.1016/j.neulet.2005.09.083.
118. López-Campos, J.L.; Silva, R.S.; Izquierdo, L.G.; Márquez, E.; Ortega, F.; Cejudo, P.; Cortés, E.B.; Aral, J.J.T.; Echevarría, M. Overexpression of Aquaporin-1 in lung adenocarcinomas and pleural mesotheliomas. *Histol. Histopathol.* 2011, *26*, 451–459,.
119. Luo, L.; Yang, R.; Zhao, S.; Chen, Y.; Hong, S.; Wang, K.; Wang, T.; Cheng, J.; Zhang, T.; Chen, D. Decreased miR-320 expression is associated with breast cancer progression, cell migration, and invasiveness via targeting Aquaporin 1. *Acta Biochim. et Biophys. Sin.* **2018**, *50*, 473–480, doi:10.1093/abbs/gmy023.
120. Luo, L.; Xia, H.; Shi, R.; Zeng, J.; Liu, X.; Wei, M. The association between aquaporin-1 expression, microvessel density and the clinicopathological features of hepatocellular carcinoma. *Oncol. Lett.* **2017**, *14*, 7077–7084, doi:10.3892/ol.2017.7106.
121. Lv, Y.; Huang, Q.; Dai, W.; Jie, Y.; Yu, G.; Fan, X.; Wu, A.; Miao, Q. AQP9 promotes astrocytoma cell invasion and motility via the AKT pathway. *Oncol. Lett.* **2018**, *16*, 6059–6064, doi:10.3892/ol.2018.9361.
122. Ma, B.; Xiang, Y.; Li, T.; Yu, H.-M.; Li, X. Inhibitory effect of topiramate on Lewis lung carcinoma metastasis and its relation with AQP1 water channel. *Acta Pharmacol. Sin.* 2004, *25*, 54–60.
123. Ma, J.; Zhou, C.; Yang, J.; Ding, X.; Zhu, Y.; Chen, X. Expression of AQP6 and AQP8 in epithelial ovarian tumor. *J. Mol. Histol.* **2016**, *47*, 129–134, doi:10.1007/s10735-016-9657-4.
124. Matsuo, K.; Kawano, K. Immunohistochemical distribution and morphometric analysis of aquaporin-3 in oral squamous cell carcinoma. *Int. J. Oral Maxillofac. Surg.* **2014**, *43*, 13–21, doi:10.1016/j.ijom.2013.05.022.
125. Mazal, P.R.; Susani, M.; Wrba, F.; Haitel, A. Diagnostic significance of aquaporin-1 in liver tumors. *Hum. Pathol.* **2005**, *36*, 1226–1231, doi:10.1016/j.humpath.2005.09.002.
126. McCoy, E.S.; Haas, B.R.; Sontheimer, H. Water permeability through aquaporin-4 is regulated by protein kinase C and becomes rate-limiting for glioma invasion. *Neurosci.* **2010**, *168*, 971–981, doi:10.1016/j.neuroscience.2009.09.020.
127. Miao, Z.-F.; Chang, E.E.; Tsai, F.-Y.; Yeh, S.-C.; Wu, C.-F.; Wu, K.-Y.; Wang, C.-J.; Tsou, T.-C. Increased aquaglyceroporin 9 expression disrupts arsenic resistance in human lung cancer cells. *Toxicol. Vitr.* **2009**, *23*, 209–216, doi:10.1016/j.tiv.2008.11.011.
128. Ming, L.; Ying, S.; Jian, Z.; Rong, W.; Ya, G. Expression and role of AQP1 in cervical squamous carcinoma and its precancerous lesions. *J. Med Coll. PLA* **2008**, *23*, 237–242, doi:10.1016/s1000-1948(08)60048-9.
129. Mobasheri, A.; Airley, R.; Hewitt, S.; Marples, D. Heterogeneous expression of the aquaporin 1 (AQP1) water channel in tumors of the prostate, breast, ovary, colon and lung: a study using high density multiple human tumor tissue microarrays. *Int. J. Oncol.* **2005**, *26*, 1149–1158, doi:10.3892/ijo.26.5.1149.
130. Scientific Program for 31st World Congress of Endourology & SWL Program Book. *J. Endourol.* **2013**, *27*, P1–A470, doi:10.1089/end.2013.2001.
131. Mobley, J.; Morrissey, J.; Bhayani, S.; Vemana, G.; Song, J.; Vetter, J.; Tanagho, Y.; Paradis, A.; Kalin, M.; Kharasch, E.; et al. 727 URINARY AQUAPORIN-1 AND PERILIPIN-2: POTENTIAL BIOMARKERS FOR EARLY DETECTION OF RENAL CELL CARCINOMA. *J. Urol.* **2013**, *189*, doi:10.1016/j.juro.2013.02.290.

132. Morrissey, J.J.; Kharasch, E.D. The Specificity of Urinary Aquaporin 1 and Perilipin 2 to Screen for Renal Cell Carcinoma. *J. Urol.* **2013**, *189*, 1913–1920, doi:10.1016/j.juro.2012.11.034.
133. Morrissey, J.J.; Mobley, J.; Figenshau, R.S.; Vetter, J.; Bhayani, S.; Kharasch, E.D. Urine Aquaporin 1 and Perilipin 2 Differentiate Renal Carcinomas From Other Imaged Renal Masses and Bladder and Prostate Cancer. *Mayo Clin. Proc.* **2015**, *90*, 35–42, doi:10.1016/j.mayocp.2014.10.005.
134. Morrissey, J.J.; Mobley, J.; Song, J.; Vetter, J.; Luo, J.; Bhayani, S.; Figenshau, R.S.; Kharasch, E.D. Urinary Concentrations of Aquaporin-1 and Perilipin-2 in Patients With Renal Cell Carcinoma Correlate With Tumor Size and Stage but not Grade. *Urol.* **2014**, *83*, 256.e9–256.e14, doi:10.1016/j.urology.2013.09.026.
135. Mou, K.; Chen, M.; Mao, Q.; Wang, P.; Ni, R.; Xia, X.; Liu, Y. AQP-4 in peritumoral edematous tissue is correlated with the degree of glioma and with expression of VEGF and HIF-alpha. *J. Neuro-Oncology* **2010**, *100*, 375–383, doi:10.1007/s11060-010-0205-x.
136. Nakakoshi, M.; Morishita, Y.; Usui, K.; Ohtsuki, M.; Ishibashi, K. Identification of a keratinocarcinoma cell line expressing AQP3. *Boil. Cell* **2006**, *98*, 95–100, doi:10.1042/bc20040127.
137. Nicchia, G.P.; Stigliano, C.; Sparaneo, A.; Rossi, A.; Frigeri, A.; Svelto, M. Inhibition of aquaporin-1 dependent angiogenesis impairs tumour growth in a mouse model of melanoma. *J. Mol. Med.* **2012**, *91*, 613–623, doi:10.1007/s00109-012-0977-x.
138. Niu, D.; Kondo, T.; Nakazawa, T.; Kawasaki, T.; Yamane, T.; Mochizuki, K.; Kato, Y.; Matsuzaki, T.; Takata, K.; Katoh, R. Differential Expression of Aquaporins and Its Diagnostic Utility in Thyroid Cancer. *PLOS ONE* **2012**, *7*, e40770, doi:10.1371/journal.pone.0040770.
139. Noell, S.; Fallier-Becker, P.; Mack, A.F.; Hoffmeister, M.; Beschorner, R.; Ritz, R. Water Channels Aquaporin 4 and -1 Expression in Subependymoma Depends on the Localization of the Tumors. *PLOS ONE* **2015**, *10*, e0131367, doi:10.1371/journal.pone.0131367.
140. Noell, S.; Ritz, R.; Wolburg-Buchholz, K.; Wolburg, H.; Fallier-Becker, P. An Allograft Glioma Model Reveals the Dependence of Aquaporin-4 Expression on the Brain Microenvironment. *PLOS ONE* **2012**, *7*, e36555, doi:10.1371/journal.pone.0036555.
141. Noell, S.; Wolburg-Buchholz, K.; Mack, A.F.; Ritz, R.; Tatagiba, M.; Beschorner, R.; Wolburg, H.; Fallier-Becker, P. Dynamics of expression patterns of AQP4, dystroglycan, agrin and matrix metalloproteinases in human glioblastoma. *Cell and Tissue Research* **2012**, *347*, 429–441, doi:10.1007/s00441-011-1321-4.
142. Oshio, K.; Binder, D.K.; Liang, Y.; Bollen, A.; Feuerstein, B.; Berger, M.S.; Manley, G.T. Expression of the aquaporin-1 water channel in human glial tumors. *Neurosurg.* **2005**, *56*, 375–381, doi:10.1227/01.neu.0000148904.57841.6b.
143. Otterbach, F.; Callies, R.; Adamzik, M.; Kimmig, R.; Siffert, W.; Schmid, K.W.; Bankfalvi, A. Aquaporin 1 (AQP1) expression is a novel characteristic feature of a particularly aggressive subgroup of basal-like breast carcinomas. *Breast Cancer Res. Treat.* **2009**, *120*, 67–76, doi:10.1007/s10549-009-0370-9.
144. Padma, S.; Smeltz, A.M.; Banks, P.M.; Iannitti, D.A.; McKillop, I.H. Altered aquaporin 9 expression and localization in human hepatocellular carcinoma. *HPB* **2009**, *11*, 66–74, doi:10.1111/j.1477-2574.2008.00014.x.
145. Pan, H.; Sun, C.-C.; Zhou, C.-Y.; Huang, H.-F. Expression of aquaporin-1 in normal, hyperplasic, and carcinomatous endometria. *Int. J. Gynecol. Obstet.* **2008**, *101*, 239–244, doi:10.1016/j.ijgo.2007.12.006.
146. Pan, X.; Guo, H.; Han, J.; Hao, F.; An, Y.; Xu, Y.; Xiaokaiti, Y.; Pan, Y.; Li, X.-J. Ginsenoside Rg3 attenuates cell migration via inhibition of aquaporin 1 expression in PC-3M prostate cancer cells. *Eur. J. Pharmacol.* **2012**, *683*, 27–34, doi:10.1016/j.ejphar.2012.02.040.
147. Park, J.Y.; Yoon, G. Overexpression of Aquaporin-1 is a Prognostic Factor for Biochemical Recurrence in Prostate Adenocarcinoma. *Pathol. Oncol. Res.* **2016**, *23*, 189–196, doi:10.1007/s12253-016-0145-7.
148. Pei, J.V.; Kourghi, M.; De Ieso, M.L.; Campbell, E.M.; Dorward, H.S.; Hardingham, J.E.; Yool, A.J. Differential Inhibition of Water and Ion Channel Activities of Mammalian Aquaporin-1 by Two Structurally Related Bacopaside Compounds Derived from the Medicinal Plant Bacopa monnieri. *Mol. Pharmacol.* **2016**, *90*, 496–507, doi:10.1124/mol.116.105882.
149. Peng, R.; Zhang, Y.; Zhao, G.; Li, J.; Shen, X.; Wang, J.; Sun, J. Differential regulation of the expression of aquaporins 3 and 9 by Auphen and dbcAMP in the SMMC-7721 hepatocellular carcinoma cell line. *Biotech. Histochem.* **2016**, *91*, 333–341, doi:10.3109/10520295.2016.1168525.
150. Peng, R.; Zhao, G.-X.; Li, J.; Zhang, Y.; Shen, X.-Z.; Wang, J.-Y.; Sun, J.-Y. Auphen and dibutyryl cAMP suppress growth of hepatocellular carcinoma by regulating expression of aquaporins 3 and 9in vivo. *World J. Gastroenterol.* **2016**, *22*, 3341–3354, doi:10.3748/wjg.v22.i12.3341.

151. Prata, C.; Facchini, C.; Leoncini, E.; Lenzi, M.; Maraldi, T.; Angeloni, C.; Zambonin, L.; Hrelia, S.; Fiorentini, D. Sulforaphane Modulates AQP8-Linked Redox Signalling in Leukemia Cells. *Oxidative Med. Cell. Longev.* **2018**, *2018*, 1–10, doi:10.1155/2018/4125297.
152. Pulford, E.; McEvoy, J.; Hocking, A.; Prabhakaran, S.; Griggs, K.; Klebe, S. The Effect of Aquaporin 1-Inhibition on Vasculogenic Mimicry in Malignant Mesothelioma. *Int. J. Mol. Sci.* **2017**, *18*, 2293, doi:10.3390/ijms18112293.
153. Qin, F.; Zhang, H.; Shao, Y.; Liu, X.; Yang, L.; Huang, Y.; Fu, L.; Gu, F.; Ma, Y. Expression of aquaporin1, a water channel protein, in cytoplasm is negatively correlated with prognosis of breast cancer patients. *Oncotarget* **2016**, *7*, 8143–8154, doi:10.18632/oncotarget.6994.
154. Qiu, J.; Zhang, Y.; Chen, H.; Guo, Z. MicroRNA-488 inhibits proliferation, invasion and EMT in osteosarcoma cell lines by targeting aquaporin 3. *Int. J. Oncol.* **2018**, *53*, 1493–1504, doi:10.3892/ijo.2018.4483.
155. Rentsch, C.A.; Bachmann, A. Editorial Comment on: Expression of Aquaporin 1 in Primary Renal Tumors: A Prognostic Indicator of Clear-Cell Renal Cell Carcinoma. *Eur. Urol.* **2009**, *56*, 699, doi:10.1016/j.eururo.2008.10.015.
156. Rouzaire-Dubois, B.; Ouanounou, G.; O'Regan, S.; Dubois, J.-M. Sodium-dependent activity of aquaporin-1 in rat glioma cells: a new mechanism of cell volume regulation. *Pflügers Archiv - European Journal of Physiology* **2008**, *457*, 1187–1198, doi:10.1007/s00424-008-0585-3.
157. Rubenwolf, P.; Thomas, C.; Denzinger, S.; Hartmann, A.; Burger, M.; Georgopoulos, N.; Otto, W. Loss of AQP3 protein expression is associated with worse progression-free and cancer-specific survival in patients with muscle-invasive bladder cancer. *World J. Urol.* **2015**, *33*, 1959–1964, doi:10.1007/s00345-015-1574-8.
158. Rubenwolf, P.; Otto, W.; Denzinger, S.; Hofstädter, F.; Wieland, W.; Georgopoulos, N. Expression of aquaporin water channels in human urothelial carcinoma: correlation of AQP3 expression with tumour grade and stage. *World J. Urol.* **2013**, *32*, 991–997, doi:10.1007/s00345-013-1153-9.
159. Saadoun, S.; Papadopoulos, M.C.; Davies, D.C.; A Bell, B.; Krishna, S. Increased aquaporin 1 water channel expression inhuman brain tumours. *Br. J. Cancer* **2002**, *87*, 621–623, doi:10.1038/sj.bjc.6600512.
160. Saadoun, S.; Papadopoulos, M.C.; Davies, D.C.; Krishna, S.; Bell, B. Aquaporin-4 expression is increased in oedematous human brain tumours. *Journal of Neurology, Neurosurgery, and Psychiatry* **2002**, *72*, 262–265, doi:10.1136/jnnp.72.2.262.
161. Saito, Y.; Furukawa, T.; Obata, T.; Saga, T. Molecular Imaging of Aquaglycero-Aquaporins: Its Potential for Cancer Characterization. *Boil. Pharm. Bull.* **2013**, *36*, 1292–1298, doi:10.1248/bpb.b13-00079.
162. Sato, K.; Miyamoto, M.; Takano, M.; Furuya, K.; Tsuda, H. Different Prognostic Implications of Aquaporin-1 and Aquaporin-5 Expression among Different Histological Types of Ovarian Carcinoma. *Pathol. Oncol. Res.* **2018**, *26*, 263–271, doi:10.1007/s12253-018-0456-y.
163. Satooka, H.; Hara-Chikuma, M. Aquaporin-3 Controls Breast Cancer Cell Migration by Regulating Hydrogen Peroxide Transport and Its Downstream Cell Signaling. *Mol. Cell. Boil.* **2016**, *36*, 1206–1218, doi:10.1128/mcb.00971-15.
164. Sawada, T.; Kato, Y.; Kobayashi, M. Expression of aquaporine-4 in central nervous system tumors. *Brain Tumor Pathol.* **2007**, *24*, 81–84, doi:10.1007/s10014-007-0216-2.
165. Schob, S.; Surov, A.; Wienke, A.; Meyer, H.J.; Spielmann, R.P.; Fiedler, E. Correlation Between Aquaporin 4 Expression and Different DWI Parameters in Grade I Meningioma. *Mol. Imaging Boil.* **2016**, *19*, 138–142, doi:10.1007/s11307-016-0978-1.
166. Sekine, S.; Okumura, T.; Nagata, T.; Shibuya, K.; Yoshioka, I.; Matsui, K.; Hori, R.; Tsukada, K. Expression Analysis of Aquaporin-1 (Aqp-1) in Human Biliary Tract Carcinoma. *J. Cancer Ther.* **2016**, *7*, 17–23, doi:10.4236/jct.2016.71003.
167. Sekine, S.; Shimada, Y.; Nagata, T.; Moriyama, M.; Omura, T.; Watanabe, T.; Hori, R.; Yoshioka, I.; Okumura, T.; Sawada, S.; et al. Prognostic significance of aquaporins in human biliary tract carcinoma. *Oncol. Rep.* **2012**, *27*, 1741–1747, doi:10.3892/or.2012.1747.
168. Sekine, S.; Shimada, Y.; Nagata, T.; Sawada, S.; Yoshioka, I.; Matsui, K.; Moriyama, M.; Omura, T.; Osawa, S.; Shibuya, K.; et al. Role of Aquaporin-5 in Gallbladder Carcinoma. *Eur. Surg. Res.* **2013**, *51*, 108–117, doi:10.1159/000355675.

169. Seleit, I.; Bakry, O.; Al Sharaky, D.; Ragheb, E. Evaluation of Aquaporin-3 Role in Nonmelanoma Skin Cancer: An Immunohistochemical Study*. *Ultrastruct. Pathol.* **2015**, *39*, 1–12, doi:10.3109/01913123.2015.1022241.
170. Shan, T.; Cui, X.; Li, W.; Lin, W.; Li, Y. AQP5: a novel biomarker that predicts poor clinical outcome in colorectal cancer. *Oncol. Rep.* **2014**, *32*, 1564–1570, doi:10.3892/or.2014.3377.
171. Shan, T.; Zheng, B.; Chen, X.; Wu, T.; Ji, E.L.; Bai, Y.H.; Wang, J.X.; Xiao, X.L. Expression of AQP5 in colorectal cancer and its relationship with clinical outcome. Journal of Xi'an Jiaotong University (Medical Sciences) 2015, 36, 815-818 and 853, doi:10.7652/jdyxb201506020.
172. Shen, Q.; Lin, W.; Luo, H.; Zhao, C.; Cheng, H.; Jiang, W.; Zhu, X. Differential Expression of Aquaporins in Cervical Precursor Lesions and Invasive Cervical Cancer. *Reprod. Sci.* **2016**, *23*, 1551–1558, doi:10.1177/1933719116646202.
173. Shi, X.; Wu, S.; Yang, Y.; Tang, L.; Lü, B. Silencing AQP-5 on proliferation, apoptosis and chemosensitivity of human colon cancer HT-29 cells. Chinese Journal of Cancer Biotherapy 2013, 20, 306-311, doi:10.3872/j.issn.1007-385X.2013.03.009.
174. Shi, X.; Wu, S.; Yang, Y.; Tang, L.; Wang, Y.; Dong, J.; Lü, B.; Jiang, G.; Zhao, W. AQP5 silencing suppresses p38 MAPK signaling and improves drug resistance in colon cancer cells. *Tumor Boil.* **2014**, *35*, 7035–7045, doi:10.1007/s13277-014-1956-3.
175. Shi, X.M.; Wu, S.C.; Tang, L.; Yang, Y.B.; Lü, B.N. Effect of AQP-5-siRNA on the apoptosis of colon cancer cell line HT-29 cells and investigation for its mechanism. Chinese Journal of Cancer Prevention and Treatment 2015, 22, 349-353.
176. Shi, Y.-H.; Chen, R.; Talafu, T.; Nijiati, R.; Lalai, S. Significance and expression of aquaporin 1, 3, 8 in cervical carcinoma in Xinjiang Uygur women of China. *Asian Pac. J. Cancer Prev.* **2012**, *13*, 1971–1975, doi:10.7314/apjcp.2012.13.5.1971.
177. Shi, Y.-H.; Rehemu, N.; Ma, H.; Tuokan, T.; Chen, R.; Suzuke, L. Increased migration and local invasion potential of SiHa cervical cancer cells expressing Aquaporin 8. *Asian Pac. J. Cancer Prev.* **2013**, *14*, 1825–1828, doi:10.7314/apjcp.2013.14.3.1825.
178. Shi, Y.H.; Tuokan, T.; Lin, C.; Chang, H. Aquaporin 8 involvement in human cervical cancer SiHa migration via the EGFR-Erk1/2 pathway. Asian Pacific journal of cancer prevention : APJCP 2014, 15, 6391-6395.
179. Shi, Z.; Zhang, T.; Luo, L.; Zhao, H.; Cheng, J.; Xiang, J.; Zhao, C. Aquaporins in human breast cancer: Identification and involvement in carcinogenesis of breast cancer. *J. Surg. Oncol.* **2011**, *106*, 267–272, doi:10.1002/jso.22155.
180. Machida, Y.; Ueda, Y.; Shimasaki, M.; Sato, K.; Sagawa, M.; Katsuda, S.; Sakuma, T. Relationship of aquaporin 1, 3, and 5 expression in lung cancer cells to cellular differentiation, invasive growth, and metastasis potential. *Hum. Pathol.* **2011**, *42*, 669–678, doi:10.1016/j.humpath.2010.07.022.
181. Shimizu, H.; Shiozaki, A.; Ichikawa, D.; Fujiwara, H.; Konishi, H.; Ishii, H.; Komatsu, S.; Kubota, T.; Okamoto, K.; Kishimoto, M.; et al. The expression and role of Aquaporin 5 in esophageal squamous cell carcinoma. *J. Gastroenterol.* **2013**, *49*, 655–666, doi:10.1007/s00535-013-0827-9.
182. Simone, L.; Gargano, C.D.; Pisani, F.; Cibelli, A.; Mola, M.G.; Frigeri, A.; Svelto, M.; Nicchia, G.P. Aquaporin-1 inhibition reduces metastatic formation in a mouse model of melanoma. *J. Cell. Mol. Med.* **2017**, *22*, 904–912, doi:10.1111/jcmm.13378.
183. Smith, E.; Palethorpe, H.M.; Tomita, Y.; Pei, J.V.; Townsend, A.R.; Price, T.J.; Young, J.; Yool, A.J.; Hardingham, J.E. The Purified Extract from the Medicinal Plant Bacopa monnieri, Bacopaside II, Inhibits Growth of Colon Cancer Cells In Vitro by Inducing Cell Cycle Arrest and Apoptosis. *Cells* **2018**, *7*, 81, doi:10.3390/cells7070081.
184. Smith, E.; Tomita, Y.; Palethorpe, H.M.; Howell, S.; Nakhjavani, M.; Townsend, A.R.; Price, T.J.; Young, J.P.; Hardingham, J.E. Reduced aquaporin-1 transcript expression in colorectal carcinoma is associated with promoter hypermethylation. *Epigenetics* **2019**, *14*, 158–170, doi:10.1080/15592294.2019.1580112.
185. Song, T.; Yang, H.; Ho, J.C.-M.; Tang, S.C.W.; Sze, S.C.W.; Lao, L.-X.; Wang, Y.; Zhang, K.Y. Expression of aquaporin 5 in primary carcinoma and lymph node metastatic carcinoma of non-small cell lung cancer. *Oncol. Lett.* **2015**, *9*, 2799–2804, doi:10.3892/ol.2015.3108.
186. Sreedharan, S.; Petros, J.A.; Master, V.A.; Ogan, K.; Pattaras, J.G.; Roberts, D.L.; Lian, F.; Arnold, R.S. Aquaporin-1 Protein Levels Elevated in Fresh Urine of Renal Cell Carcinoma Patients: Potential Use for

Screening and Classification of Incidental Renal Lesions. *Dis. Markers* **2014**, *2014*, 1–6, doi:10.1155/2014/135649.

187. Kang, S.K.; Chae, Y.K.; Woo, J.; Kim, M.S.; Park, J.C.; Lee, J.; Soria, J.C.; Jang, S.J.; Sidransky, D.; Moon, C. Role of Human Aquaporin 5 In Colorectal Carcinogenesis. *Am. J. Pathol.* **2008**, *173*, 518–525, doi:10.2353/ajpath.2008.071198.
188. Thapa, S.; Chetry, M.; Huang, K.; Peng, Y.; Wang, J.; Wang, J.; Zhou, Y.; Shen, Y.; Xue, Y.; Ji, K. Significance of aquaporins' expression in the prognosis of gastric cancer. *Biosci. Rep.* **2018**, *38*, 38,, doi:10.1042/bsr20171687.
189. Ticozzi-Valerio, D.; Raimondo, F.; Pitto, M.; Rocco, F.; Bosari, S.; Perego, R.; Sarto, C.; Di Fonzo, A.; Bosso, N.; Mocarelli, P.; et al. Differential expression of AQP1 in microdomain-enriched membranes of renal cell carcinoma. *Proteom. - Clin. Appl.* **2007**, *1*, 588–597, doi:10.1002/prca.200601048.
190. Vacca, A.; Frigeri, A.; Ribatti, D.; Nicchia, G.P.; Nico, B.; Ria, R.; Svelto, M.; Dammacco, F. Microvessel overexpression of aquaporin 1 parallels bone marrow angiogenesis in patients with active multiple myeloma. *Br. J. Haematol.* **2001**, *113*, 415–421, doi:10.1046/j.1365-2141.2001.02738.x.
191. Wang, D.; Owler, B.K. Expression of AQP1 and AQP4 in paediatric brain tumours. *J. Clin. Neurosci.* **2011**, *18*, 122–127, doi:10.1016/j.jocn.2010.07.115.
192. Warth, A.; Muley, T.R.; Meister, M.; Herpel, E.; Pathil-Warth, A.; Hoffmann, H.; A Schnabel, P.; Bender, C.; Buneß, A.; Schirmacher, P.; et al. Loss of aquaporin-4 expression and putative function in non-small cell lung cancer. *BMC Cancer* **2011**, *11*, 161, doi:10.1186/1471-2407-11-161.
193. Pathil-Warth, A.; Simon, P.; Capper, D.; Goeppert, B.; Tabatabai, G.; Herzog, H.; Dietz, K.; Stubenvoll, F.; Ajaaj, R.; Becker, R.; et al. Expression pattern of the water channel aquaporin-4 in human gliomas is associated with blood–brain barrier disturbance but not with patient survival. *J. Neurosci. Res.* **2007**, *85*, 1336–1346, doi:10.1002/jnr.21224.
194. Wu, D.Q.; Yang, Z.F.; Wang, K.J.; Feng, X.Y.; Lv, Z.J.; Li, Y.; Jian, Z.X. AQP8 inhibits colorectal cancer growth and metastasis by down-regulating PI3K/AKT signaling and PCDH7 expression. *Am. J. Cancer Res.* 2018, *8*, 266–279.
195. Xia, H.; Ye, J.; Bai, H.; Wang, C. Effects of cetuximab combined with celecoxib on apoptosis and KDR and AQP1 expression in lung cancer. Chinese Journal of Lung Cancer 2013, 16, 625-631, doi:10.3779/j.issn.1009-3419.2013.12.02.
196. Xiong, W.; Ran, J.; Jiang, R.; Guo, P.; Shi, X.; Li, H.; Lv, X.; Li, J.; Chen, D. miRNA-320a inhibits glioma cell invasion and migration by directly targeting aquaporin 4. *Oncol. Rep.* **2018**, *39*, 1939–1947, doi:10.3892/or.2018.6274.
197. Xuejun, C.; Weimin, C.; Xiaoyan, D.; Wei, Z.; Qiong, Z.; Jianhua, Y. Effects of aquaporins on chemosensitivity to cisplatin in ovarian cancer cells. *Arch. Gynecol. Obstet.* **2014**, *290*, 525–532, doi:10.1007/s00404-014-3216-6.
198. Yang, C.; Lim, W.; Bae, H.; Song, G.; Information, P.E.K.F.C. Aquaporin 3 is regulated by estrogen in the chicken oviduct and is involved in progression of epithelial cell–derived ovarian carcinomas. *Domest. Anim. Endocrinol.* **2016**, *55*, 97–106, doi:10.1016/j.domaniend.2015.12.003.
199. Yang, J.; Yan, C.; Zheng, W.; Chen, X. Proliferation inhibition of cisplatin and aquaporin 5 expression in human ovarian cancer cell CAOV3. *Arch. Gynecol. Obstet.* **2011**, *285*, 239–245, doi:10.1007/s00404-011-1908-8.
200. Yang, J.; Zhang, J.-N.; Chen, W.-L.; Wang, G.-S.; Mao, Q.; Li, S.-Q.; Xiong, W.-H.; Lin, Y.-Y.; Ge, J.-W.; Li, X.-X.; et al. Effects of AQP5 gene silencing on proliferation, migration and apoptosis of human glioma cells through regulating EGFR/ERK/ p38 MAPK signaling pathway. *Oncotarget* **2017**, *8*, 38444–38455, doi:10.18632/oncotarget.16461.
201. Yang, J.; Shi, Y.; Chen, X.; Qi, W. The influence of aquaporin-1 and microvessel density on ovarian carcinogenesis and ascites formation. *Int. J. Gynecol. Cancer* **2006**, *16*, 400–405, doi:10.1111/j.1525-1438.2006.00476.x.
202. Yang, J.-H.; Shi, Y.-F.; Cheng, Q.; Deng, L. Expression and localization of aquaporin-5 in the epithelial ovarian tumors. *Gynecol. Oncol.* **2006**, *100*, 294–299, doi:10.1016/j.ygyno.2005.08.054.
203. Yang, J.H.; Yan, C.X.; Chen, X.J.; Zhu, Y.S. Expression of aquaglyceroporins in epithelial ovarian tumours and their clinical significance. *J. Int. Med Res.* **2011**, *39*, 702–711, doi:10.1177/147323001103900302.
204. Yang, J.; Yu, Y.-Q.; Yan, C. Localisation and expression of aquaporin subtypes in epithelial ovarian tumours. *Histol. Histopathol.* 2011, *26*, 1197–1205,.

205. Yang, L.; Wang, X.; Zhen, S.; Zhang, S.; Kang, D.; Lin, Z. Aquaporin-4 upregulated expression in glioma tissue is a reaction to glioma-associated edema induced by vascular endothelial growth factor. *Oncol. Rep.* **2012**, *28*, 1633–1638, doi:10.3892/or.2012.1973.
206. Yang, W.-C.; Zhou, L.-J.; Zhang, R.; Yue, Z.-Y.; Dong, H.; Song, C.-Y.; Qian, H.; Lü, S.-J.; Chang, F.-F. Effects of propofol and sevoflurane on aquaporin-4 and aquaporin-9 expression in patients performed gliomas resection. *Brain Res.* **2015**, *1622*, 1–6, doi:10.1016/j.brainres.2015.05.042.
207. Yang, Z.-H.; Feng, X.-Z.; Huang, D.-D.; Deng, Y.-H.; Chen, H.; Wang, J. Sa1929 Aquaporin-9 Overexpression Activates Akt Signal Pathway and Enhances Chemosensitivity in Colorectal Cancer. *Gastroenterol.* **2015**, *148*, 357-, doi:10.1016/s0016-5085(15)31203-8.
208. Yin, T.; Yu, S.; Xiao, L.; Zhang, J.; Liu, C.; Lu, Y.; Liu, C. Correlation between the expression of aquaporin 1 and hypoxia-inducible factor 1 in breast cancer tissues. *Acta Acad. Med. Wuhan* **2008**, *28*, 346–348, doi:10.1007/s11596-008-0327-y.
209. Jiang, Y. Aquaporin-1 activity of plasma membrane affects HT20 colon cancer cell migration. *IUBMB Life* **2009**, *61*, 1001–1009, doi:10.1002/iub.243.
210. Nico, B.; Ribatti, D. Role of aquaporins in cell migration and edema formation in human brain tumors. *Exp. Cell Res.* **2011**, *317*, 2391–2396, doi:10.1016/j.yexcr.2011.07.006.
211. Betz, A.L.; Iannotti, F.; Hoff, J.T. Brain edema: a classification based on blood-brain barrier integrity. *Cerebrovasc. brain Metab. Rev.* 1989, *1*, 133–154.
212. Yoshida, T.; Hojo, S.; Sekine, S.; Sawada, S.; Okumura, T.; Nagata, T.; Shimada, Y.; Tsukada, K. Expression of aquaporin-1 is a poor prognostic factor for stage II and III colon cancer. *Mol. Clin. Oncol.* **2013**, *1*, 953–958, doi:10.3892/mco.2013.165.
213. Yun, S.; Sun, P.-L.; Jin, Y.; Kim, H.; Park, E.; Park, S.Y.; Lee, K.; Lee, K.; Chung, J.-H. Aquaporin 1 Is an Independent Marker of Poor Prognosis in Lung Adenocarcinoma. *J. Pathol. Transl. Med.* **2016**, *50*, 251–257, doi:10.4132/jptm.2016.03.30.
214. Zhang, G.; Lü, J.; Zhou, H.; Du, Z.; Zhang, G. Silencing of aquaporinï¿½5 inhibits the growth of A549 lung cancer cells in vitro and in vivo. *Int. J. Oncol.* **2018**, *52*, 1643–1650,, doi:10.3892/ijo.2018.4326.
215. Zhang, L.; Sun, H.; Xin, J.; Du, S.; Zhang, L.; Li, K. vascular endothelial growth factor, Aquaporin-1 level and clinicopathological features of cervical cancer. Chinese Journal of Medical Imaging Technology 2018, 34, 260-264, doi:10.13929/j.1003-3289.201707007.
216. Zhang, T.; Lu, X.W.; Cheng, J.; Xiang, J.Y.; Chen, D.Z. Decreased miR-320 and increased AQP1 in patients with breast cancer and the clinical significance. International Journal of Gynecological Cancer 2013, 23, 678.
217. Zhang, T.; Zhao, C.; Chen, D.; Zhou, Z. Overexpression of AQP5 in cervical cancer: correlation with clinicopathological features and prognosis. *Med Oncol.* **2011**, *29*, 1998–2004, doi:10.1007/s12032-011-0095-6.
218. Zhang, W.-G.; Li, C.-F.; Liu, M.; Chen, X.-F.; Shuai, K.; Kong, X.; Lv, L.; Mei, Z.-C.; Kong, X. Aquaporin 9 is down-regulated in hepatocellular carcinoma and its over-expression suppresses hepatoma cell invasion through inhibiting epithelial-to-mesenchymal transition. *Cancer Lett.* **2016**, *378*, 111–119, doi:10.1016/j.canlet.2016.05.021.
219. Zhang, X.; Chen, Y.; Dong, L.; Shi, B. Effect of selective inhibition of aquaporin 1 on chemotherapy sensitivity of J82 human bladder cancer cells. *Oncol. Lett.* **2018**, *15*, 3864–3869, doi:10.3892/ol.2018.7727.
220. Zhang, Z.; Chen, Z.; Song, Y.; Zhang, P.; Hu, J.; Bai, C. Expression of aquaporin 5 increases proliferation and metastasis potential of lung cancer. *J. Pathol.* **2010**, *221*, 210–220, doi:10.1002/path.2702.
221. Zhang, Z.; Han, Y.; Sun, G.; Liu, X.; Jia, X.; Yu, X. MicroRNA-325-3p inhibits cell proliferation and induces apoptosis in hepatitis B virus-related hepatocellular carcinoma by down-regulation of aquaporin 5. *Cell. Mol. Boil. Lett.* **2019**, *24*, 1–15, doi:10.1186/s11658-019-0137-1.
222. Zhang, Z.-Q.; Zhu, Z.-X.; Bai, C.; Chen, Z. Aquaporin 5 expression increases mucin production in lung adenocarcinoma. *Oncol. Rep.* **2011**, *25*, 1645–1650,, doi:10.3892/or.2011.1241.
223. Zhao, W.-J.; Zhang, W.; Li, G.; Cui, Y.; Shi, Z.-F.; Yuan, F. Differential expression of MMP-9 and AQP4 in human glioma samples. *Folia Neuropathol.* 2012, *50*, 176–186.
224. Zhu, S.-J.; Wang, K.; Gan, S.-W.; Xu, J.; Xu, S.-Y.; Sun, S.-Q. Expression of aquaporin8 in human astrocytomas: Correlation with pathologic grade. *Biochem. Biophys. Res. Commun.* **2013**, *440*, 168–172, doi:10.1016/j.bbrc.2013.09.057.
225. Zou, L.; Zhang, R.-J.; Tan, Y.-J.; Ding, G.; Shi, S.; Zhang, D.; He, R.-H.; Liu, A.-X.; Wang, T.-T.; Leung, P.C.K.; et al. Identification of Estrogen Response Element in the Aquaporin-2 Gene That Mediates

Estrogen-Induced Cell Migration and Invasion in Human Endometrial Carcinoma. *J. Clin. Endocrinol. Metab.* **2011**, *96*, 1399–1408,, doi:10.1210/jc.2011-0426.
226. Zou, W.; Yang, Z.; Li, D.; Liu, Z.; Zou, Q.; Yuan, Y. AQP1 and AQP3 Expression are Associated With Severe Symptoms and Poor-prognosis of the Pancreatic Ductal Adenocarcinoma. *Appl. Immunohistochem. Mol. Morphol.* **2019**, *27*, 40–47, doi:10.1097/pai.0000000000000523.

© 2020 by the authors. Licensee MDPI, Basel, Switzerland. This article is an open access article distributed under the terms and conditions of the Creative Commons Attribution (CC BY) license (http://creativecommons.org/licenses/by/4.0/).
